# Atlantic meridional overturning circulation slowdown modulates atmospheric rivers in a warmer climate

**DOI:** 10.1038/s41467-026-72555-w

**Published:** 2026-05-04

**Authors:** Mohima Sultana Mimi, Wei Liu, Weiming Ma, Gang Chen

**Affiliations:** 1 Department of Earth and Planetary Sciences, University of California Riverside, Riverside, CA USA; 2 Atmospheric, Climate, and Earth Sciences Division, Pacific Northwest National Laboratory, Richland, WA USA; 3 Department of Atmospheric and Oceanic Sciences, University of California Los Angeles, Los Angeles, CA USA

**Keywords:** Atmospheric science, Ocean sciences, Projection and prediction

## Abstract

The slowing of the Atlantic meridional overturning circulation (AMOC) under anthropogenic warming has been suggested to significantly impact Earth’s climate. Here, we isolate and quantify the AMOC impact on atmospheric rivers (ARs) across the twenty-first century using coupled climate model simulations. We find that a weakened AMOC promotes AR frequency in mid-latitudes by intensifying the prevailing westerly winds, especially at the west coast of North America, which dramatically enhances AR-induced precipitation in wintertime California. Aside from dynamic processes, the weakened AMOC can also modulate ARs through thermodynamic processes. It reduces AR frequency and related precipitation over the Arctic and Greenland while increasing AR frequency and associated precipitation along the eastern coast of South America and around Antarctica, owing primarily to AMOC-induced moisture decrease and increase in the Northern and Southern Hemispheres, respectively. Our findings highlight the role of the AMOC in future regional hydroclimate and climate extreme shift.

## Abstract (web summary)

Using twenty-first-century climate model projections, this study shows that a weakening of the Atlantic meridional overturning circulation will increase the frequency of atmospheric rivers in the mid-latitudes, especially along North America’s west coast, leading to significant increase of atmospheric river–driven winter precipitation in California.

## Introduction

Atmospheric rivers (ARs) are long, narrow corridors of intense water vapor transport in the lower troposphere, typically associated with low-level jet streams ahead of the cold fronts of extratropical cyclones^1–4^. They cover only about 10% of the Earth’s mid-latitude zonal circumference but serve as an essential component of global hydrological cycle, responsible for nearly 90% of poleward moisture transport in the mid-latitudes^4–8^. ARs can strongly impact regional climate. For instance, they are a major driver of both water resources and flood hazards in the west coast of United States^5,9,10^. In the Arctic, ARs contribute to surface warming and sea ice loss^11–14^. Over Antarctica, ARs account for 40–80% of summer meltwater in West Antarctic ice shelves, which threatens ice stability and accelerates global sea level rise^15,16^.

ARs are subject to alter under climate change^17,18^. By the end of the twenty-first century, global mean AR frequency is projected to increase by ~50% under the Representative Concentration Pathway 8.5 (RCP8.5) scenario^19^, with more pronounced increases in the southern and northern mid-latitudes. Stronger ARs (categories 3–5 on the Ralph scale^7^) are expected to become more common, with ARs penetrating higher latitudes as the westerly jet shifts poleward under anthropogenic warming^2,20–22^. Mechanisms driving the AR change have been extensively explored, including internal climate variability^14^ and anthropogenic aerosol and greenhouse gas forcings^23^. Nevertheless, the impact of the Atlantic meridional overturning circulation (AMOC), a key player in the Earth’s climate system, on ARs, has seldom been discussed.

The AMOC has exhibited a declining trend since the 1990s^24^. In addition to recent measurements from the Rapid Climate Change-Meridional Overturning Circulation and Heatflux Array (RAPID) at 26.5°N in the North Atlantic^25^, proxy reconstructions indicate that the AMOC began to slow down during the mid-to-late twentieth century^26–29^. This slowdown is projected to continue throughout the twenty-first century under high-emission scenarios such as the RCP8.5 and Shared Socioeconomic Pathway 5-8.5 (SSP5-8.5)^30,31^. The ongoing and projected declines of the AMOC could profoundly affect various elements in the Earth’s climate system^32–37^, for example, modifying the pattern of sea-surface-temperature change^32,38^, displacing the tropical rain belt southward^39^ and increasing rainfall in parts of the Amazon rainforest^40^, modulating the North Atlantic storm track and moisture transports^32,41,42^, all of which will potentially influence ARs. Nevertheless, such AMOC effect on ARs and associated mechanisms remain unclear, owing primarily to concurrent changes in the AMOC and ARs in response to anthropogenic forcing, as well as the difficulty to distinguish the effect of AMOC slowing from the influences of other varying and interacting climatic components within a fully coupled climate system. To address this knowledge gap, we employ reanalysis data and coupled climate model simulations to investigate how AMOC weakening impact global and regional ARs and to elucidate the underlying physical processes.

## Results

### AR and AMOC during past decades

**Fig. 1:**
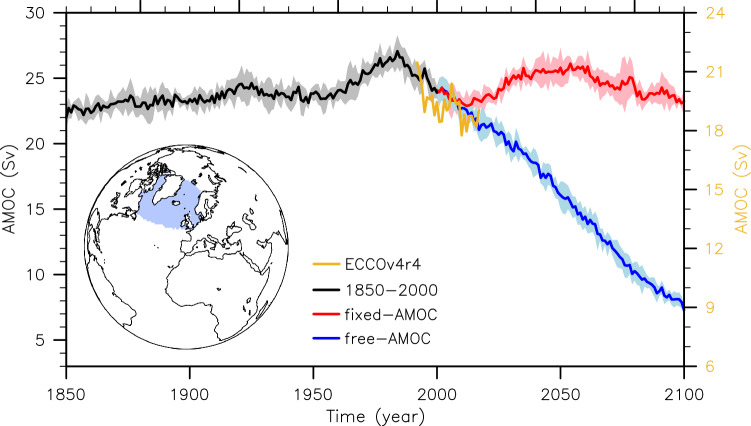
Atlantic Meridional Overturning Circulation (AMOC) in reanalysis and model simulation. AMOC strength (Methods) derived from ECCOv4r4 reanalysis (1992–2017; orange) and CESM2 historical plus SSP5-8.5 simulation (denoted as free-AMOC; prior to 2000, ensemble mean, black; ensemble spread, gray; after 2000, ensemble mean, blue; ensemble spread, light blue) and CESM2 fixed-AMOC experiment (denoted as fixed-AMOC; ensemble mean, red; ensemble spread, light red) where ensemble spread represents one standard derivation among ensembles. CESM2 and reanalysis AMOC strengths correspond to the left and right axes, respectively. The inserted map shows the freshwater removal region in the CESM2 fixed-AMOC experiment (light blue shading). The southern boundary of the region is along 50°N, and the northern boundary is close to 80°N, which separates the Arctic in the north along the Canadian Arctic Archipelago, the Fram Strait, and the western shelf of the Barents Sea. The base map is from NCAR Command Language map outline databases.

We show the evolution of ARs since the 1990s from the NASA Modern-Era Retrospective Analysis for Research and Applications version 2 (MERRA-2; Methods). We calculate AR frequency based on integrated vapor transport (IVT; Methods) wherein AR frequency is defined as the fraction of time a grid point experiences AR conditions. During 1992-2014, ARs are most active in the mid-latitudes, with an annual frequency of 9.04%, substantially higher than those in the tropics (2.74%) and polar (3.89%) regions (Supplementary Fig. 1a), as consistent with previous studies^4–8,43^. We also examine the AMOC strength from the Estimating the Circulation and Climate of the Ocean version 4 release 4 (ECCOv4r4; Methods), which depicts an active AMOC with an average strength around 19.32 Sv (1 Sv = 10^6^ m^3^s^-1^) from 1992 to 2014 (Fig. 1, yellow) as consistent with the RAPID observation^25^. It is noteworthy that the AMOC shows a significant slowing trend of -0.80 Sv/decade (*p* < 0.001, Methods) between 1992 and 2014, notwithstanding decadal variability. These characteristics of ARs (Supplementary Fig. 1b) and the AMOC (Fig. 1, black and blue) are well simulated by a broadly used climate model, Community Earth System Model version 2 (CESM2)^44^ (Methods), justifying the usage of the model for further investigation of the AMOC impact on ARs under future climate change.

### AMOC impact on AR frequency

We harness CESM2 ensemble historical and SSP5-8.5 simulations (referred to as the free-AMOC scenario), which illustrate a noticeable slowdown of the AMOC from the 1980s to 2100 (Fig. 1, black and blue). Building on this free-AMOC framework, we conduct a parallel ensemble simulation (Methods) that maintains a fixed-AMOC strength under the SSP5-8.5 scenario throughout the twenty-first century (Fig. 1, red). This fixed-AMOC experiment is driven by the same historical and SSP5-8.5 forcing agents as the free-AMOC simulation, but it involves the gradual removal of freshwater from the surface in deep-water formation regions over the North Atlantic (Fig. 1, the inserted map, blue shaded), which is then uniformly redistributed across the global oceans^31^. This method allows for natural atmosphere-ocean interactions in a fully coupled climate system. The difference between the free- and fixed-AMOC simulations elucidates the impact of AMOC slowdown on ARs.

**Fig. 2:**
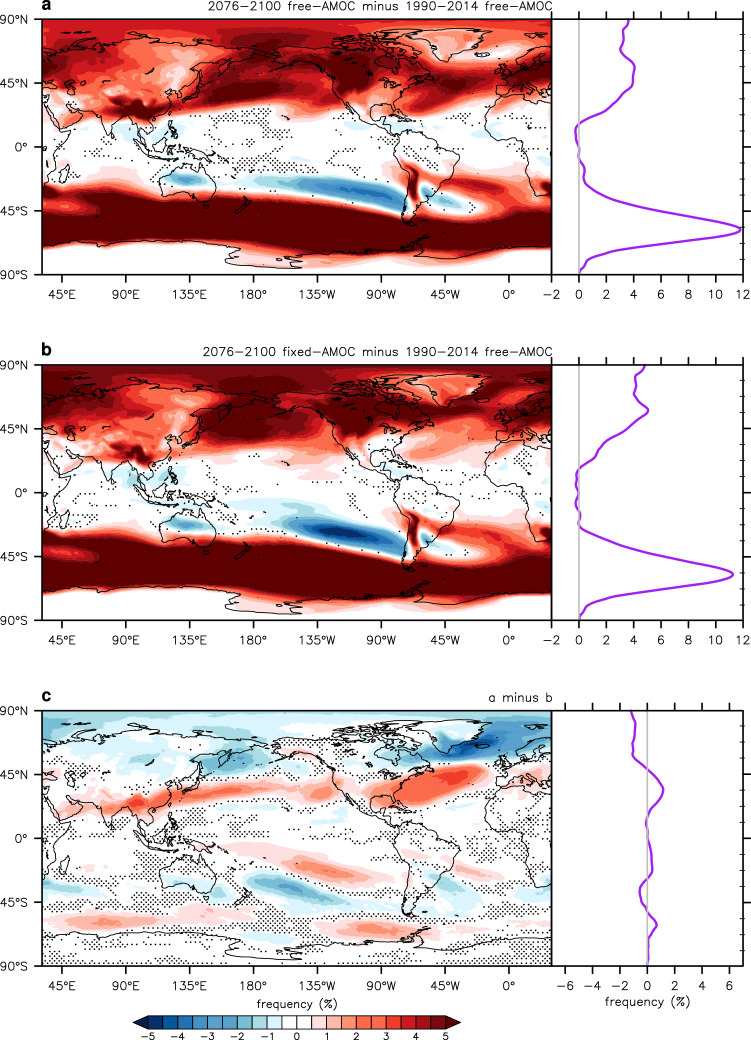
Global Atmospheric River (AR) frequency change and Atlantic Meridional Overturning Circulation (AMOC) impact. Changes in annual mean AR frequency (shading in %) for the ensemble means of CESM2 (**a**) free- and (**b**) fixed-AMOC simulations during the period of 2076–2100 relative to 1990–2014, and (**c**) the difference between the two (**a** minus **b**), along with the zonal mean of AR frequency (%), respectively. The stipples refer to the regions where changes are not significantly different from zero at the 95% confidence level of the Student’s t-test. The base map is from NCAR Command Language map outline databases.

We first look at the ensemble mean of the free-AMOC simulation, focusing on the AR change toward the end of the twenty-first century. Compared to 1990–2014, ARs will become more frequent over the mid-to-high latitudes during 2076–2100 (Fig. 2a). The zonal mean profile of AR frequency reveals that AR frequency increases by 3.94% at 45^o^N, by 3.28% on average over the Arctic region (60^o^N–90^o^N) and by up to 11.79% at around 45^o^S over the Southern Ocean. Meanwhile, ARs will become less frequent in lower latitudes, for example, over northern Australia (up to −2.26%) and the southeast South Pacific (up to −3.56%). The changes in AR frequency align with earlier findings^7,15,16,19,45^. Nonetheless, if the AMOC were not to slow, the AR frequency pattern will be markedly modulated, especially in the Northern Hemisphere (Fig. 2b). We further look into the difference between the free- and fixed-AMOC simulations during 2076-2100 to determine the AMOC influence. From the zonal-mean difference, a weakened AMOC will increase AR frequency over the Northern Hemisphere subtropics to mid-latitudes and Southern Hemisphere subtropics and high-latitudes while decreasing it over the Northern Hemisphere mid-to-high latitudes and Southern Hemisphere mid-latitudes (Fig. 2c). The largest shifts in AR frequency will occur in the climatological AR peak regions, especially between 30°N and 50°N along the west coast of North America, extending from Baja California to Alaska (Fig. 2c). The slowed AMOC will give rise to more AR landfall on the west coast of the United States, which potentially modulates the hydroclimate in California and Arizona regions^9,46–48^. This AMOC impact on AR landfall along the west coast of North America is particularly prominent during the boreal winter (up to +5.21%) (Supplementary Fig. 2c). In contrast, the AMOC slowing will bring about a significant decline in AR frequency over the Arctic regions, including Greenland and northern Asia (Fig. 2c), which mainly occurs during boreal winters (up to −9.21%) rather than boreal summers (Supplementary Figs. 2c and 3c).

Over the North Atlantic, the AMOC slowing will promote AR frequency on both side of the basin, up to +3.02% and +1.53% in the North America and Europe, respectively (Fig. 2c), which has profound implications on the hydroclimate in these two regions. This is because, under the global warming scenario, AR frequency is projected to dramatically augment over the Atlantic basin in the Northern Hemisphere, impacting the east coast of North America, Northwestern Europe, and the British Isles^49,50^. It should be noted that the Gulf region will even experience less frequent ARs if the AMOC were not to slow down (Fig. 2b), suggesting that the slowed AMOC will reverse a decline ( − 1.07%) to increase ( + 2.45%) in AR frequency in this region (Fig. 2a). On the other hand, the AMOC effect manifests an increase in AR frequency across South, Southeast and East Asia (Fig. 2c), which occurs throughout the year (Supplementary Figs. 2c and 3c).

In the Southern Hemisphere, the weakened AMOC is projected to increase the AR frequency in a band extending from the western South Pacific near the Equator to more extratropical eastern South Pacific (up to +2.09%), as well as decrease the AR frequency in a band to the southwest (up to −2.32%) (Fig. 2c). This pattern indicates a northeast displacement of AR frequency change from the fixed-AMOC case (Fig. 2b). A similar pattern also occurs in the eastern South Atlantic, meaning that the AMOC deceleration will increase the AR frequency along the coast of eastern Brazil (up to +1.27%), while decreasing it to the south (up to −1.57%). These changes are especially evident during the austral winter (Supplementary Fig. 3c). It is worth noting that the slower AMOC will raise the AR frequency over the marginal seas around Antarctica, such as the Cooperation, Davis, Amundsen, and Bellingshausen Seas (Fig. 2c), by up to +3.80% during austral winters (Supplementary Fig. 3c).

We also compare the probability density functions of AR basic characteristics between the free- and fixed-AMOC simulations during 2076-2100. The slower AMOC has little effect on the coherence of IVT direction (Supplementary Fig. 4f). It, however, raises the probability of ARs with lengths and widths less than 6000 km and 900 km, while reducing the probability otherwise (Supplementary Fig. 4a,b), implying a shift of ARs towards shorter and narrower. The slower AMOC generally increases the likelihoods of AR’s equatorward and poleward ends in the Northern Hemisphere but decreasing them in the Southern Hemisphere (Supplementary Fig. 4c and 4d), indicative of a southward shift in the AR position. Additionally, the slower AMOC diminishes and enhances the likelihood of the mean IVT in azimuths of smaller and larger than 90°, respectively (Supplementary Fig. 4e), flattens the probability peak of the mean IVT, and shifts the landfall IVT toward smaller amplitude (Supplementary Fig. 4g and 4h).

### AMOC impact on AR induced precipitation

**Fig. 3:**
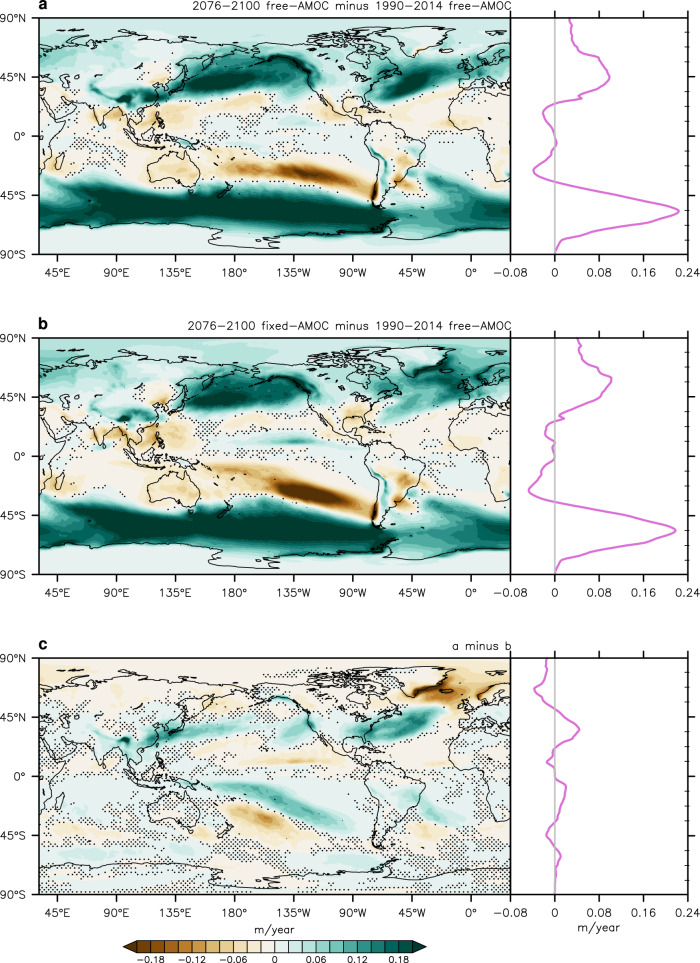
Global Atmospheric River (AR)-induced precipitation change and Atlantic Meridional Overturning Circulation (AMOC) impact. Changes in annual mean AR-induced precipitation (shading in m/year) for the ensemble means of CESM2 (**a**) free- and (**b**) fixed-AMOC simulations during the period of 2076–2100 relative to 1990–2014, and (**c**) the difference between the two (**a** minus **b**), along with the zonal mean of AR-induced precipitation (m/year), respectively. The stipples refer to the regions where changes are not significantly different from zero at the 95% confidence level of the Student’s t-test. The base map is from NCAR Command Language map outline databases.

The response of annual mean AR-induced precipitation to AMOC decline is generally in line with AR frequency response over a global scale (Fig. 3). Changes are pronounced in the mid-to-high latitudes of both hemispheres, including the southern Greenland (up to −0.44 m/year), the east (up to +0.17 m/year) and west (up to +0.16 m/year) coasts of North America, the eastern South America near Brazil (up to +0.07 m/year), South, Southeast and East Asia (up to +0.47 m/year) and the south-central Pacific (up to +0.11 m/year) (Fig. 3c). For instance, the polar regions of the Northern Hemisphere, such as the southern Greenland and the GIN (Greenland, Iceland, and Norwegian) Seas will experience similar or even less AR-induced precipitation in 2076-2100 than 1990-2014 (Fig. 3a), whereas these regions would receive more AR induced precipitation if the AMOC were not to decelerate (Fig. 3b), meaning that the weaker AMOC will diminish AR-induced precipitation in the regions by the end of the twenty-first century (Fig. 3c). This AR induced precipitation change can strongly affect extreme weather events and icesheet melt in Greenland^45,51^, as well as Arctic sea ice melt^16^.

On the other hand, coastal areas like those in California and South Carolina will experience enhanced AR-induced precipitation as a result of AMOC slowing (Fig. 3c). Compared to 1990–2014, AR-induced precipitation increase during 2076–2100 can reach 0.07 m/year for state average in California and 0.09 m/year in South Carolina in the free-AMOC case (Fig. 3a), but they will be limited to 0.01 m/year and −0.05 m/year, respectively, in the fixed-AMOC case (Fig. 3b), evincing that the weakened AMOC amplifies the California increase by more than fivefold while reversing the sign of the AR-induced precipitation response in South Carolina. The AMOC influence is especially striking in wintertime California, where the weakened AMOC will enhance AR-induced precipitation by 0.18 m/year (up to 0.44 m/year) by the end of the current century (Supplementary Figs. 5 and 6). Such an impact will be enormous, seeing that ARs produce the bulk of extreme precipitation events in California that results in either beneficial water supply or disastrous flooding^5,10,52^.

### Physical mechanisms

**Fig. 4:**
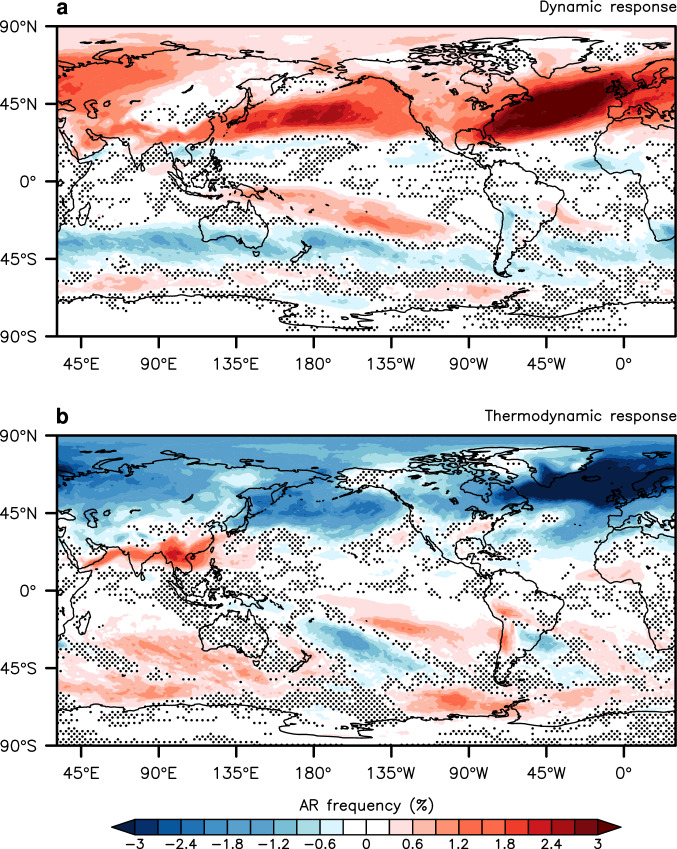
Dynamic and thermodynamic components of Atlantic Meridional Overturning Circulation (AMOC) impact on Atmospheric River (AR) frequency. **a** Dynamic and (**b**) thermodynamic contributions to annual mean AR frequency response (shading in %) to AMOC decline during 2076–2100 for CESM2 ensemble mean. Noting the different contouring scales from those in Fig. 2. The stipples refer to the regions where changes are not significantly different from zero at the 95% confidence level of the Student’s t-test. The base map is from NCAR Command Language map outline databases.

**Fig. 5:**
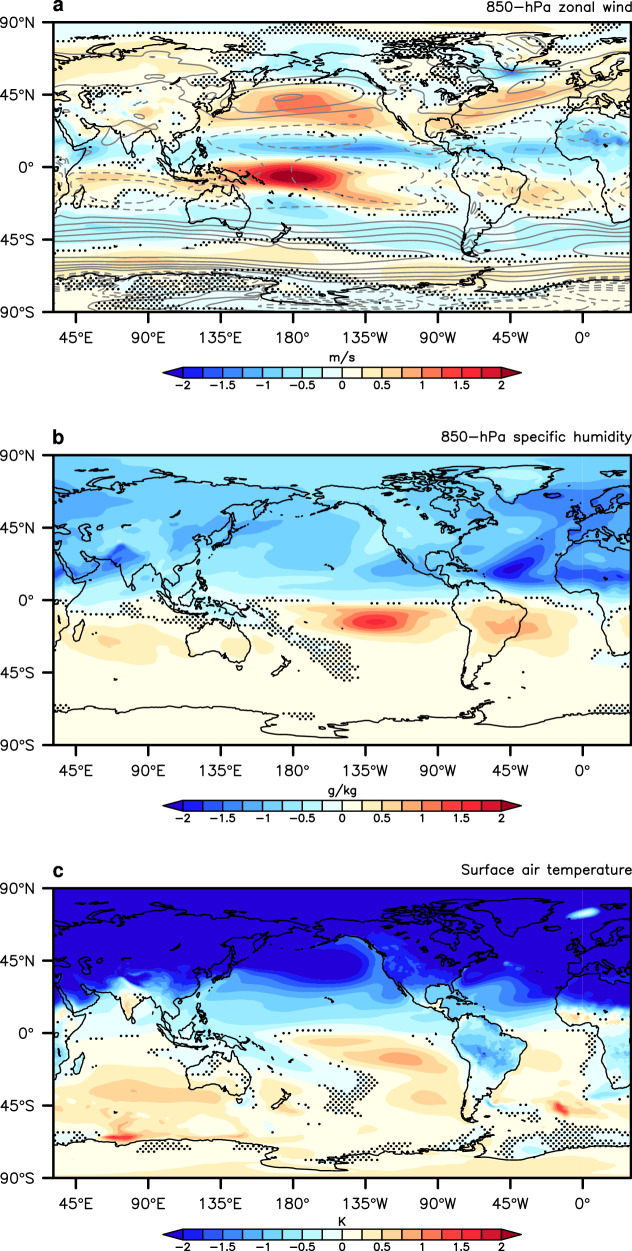
Atlantic Meridional Overturning Circulation (AMOC) impact on wind, specific humidity, and surface air temperature. **a** Annual mean 850 hPa zonal wind (shading in m/s) for the ensemble mean of CESM2 free- minus fixed-AMOC simulations during 2076-2100, with contours (in an interval of 3 m s^-1^, solid positive, dashed negative, and zero neglected) showing the free-AMOC simulation climatology during this period. **b**, **c** similar to (**a**) but for annual mean 850 hPa specific humidity (shading in g kg^-1^) and surface air temperature (shading in K), respectively. The stipples refer to the regions where changes are not significantly different from zero at the 95% confidence level of the Student’s t-test. The base map is from NCAR Command Language map outline databases.

**Fig. 6:**
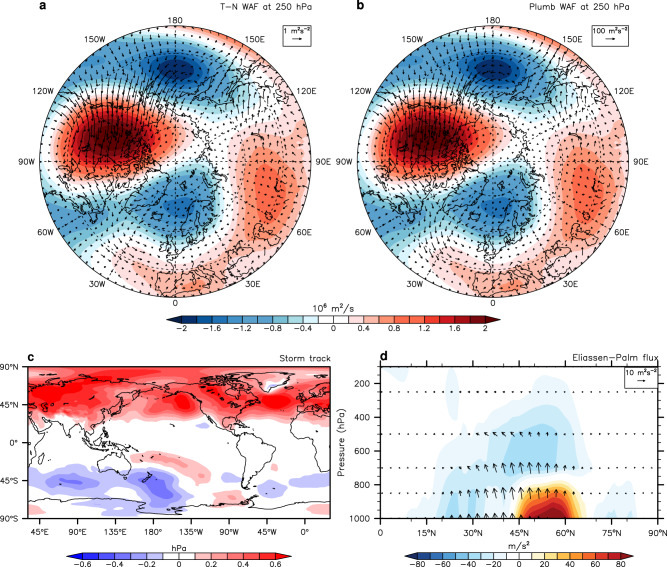
Atlantic Meridional Overturning Circulation (AMOC) impact on stationary wave-activity flux, storm-track activity and Eliassen–Palm flux. Annual mean quasi-geostrophic stream-function anomalies (shading in 10^6 ^m² s⁻¹), and horizontal (**a**) Takaya–Nakamura and (**b**) Plumb wave-activity flux anomalies (vectors in m² s⁻²) associated with a stationary Rossby wave train at 250 hPa, (**c**) Storm track (shading in hPa) inferred from the standard deviation of 2–6-day bandpass-filtered sea level pressure, (**d**) Annual mean Eliassen–Palm flux (vectors in m² s⁻²) and its divergence (shading in m s⁻^2^) for the ensemble mean of CESM2 free- minus fixed-AMOC simulation during 2076–2100. The base map is from NCAR Command Language map outline databases.

To exploit the physical mechanisms by which the AMOC influences ARs, we begin by decomposing the total AR frequency change between 2076-2100 and 1990-2014 into dynamic and thermodynamic components (Methods). The dynamic component, primarily driven by changes in atmospheric circulation, exerts the most pronounced effects in the Northern Hemisphere mid-latitudes, where westerly winds dominate (Fig. 4a). Seen from 850 hPa zonal wind response to the AMOC decline during 2076–2100, westerly jets strengthen over both North Atlantic and Pacific Oceans (Fig. 5a). Over the former ocean, the slower AMOC alters meridional SST gradients, and hence modulates the North Atlantic jets via baroclinicity, local air-sea interaction^41,53^ and eddy-mean flow interaction^32,54^. Meanwhile, the AMOC-induced surface cooling in the North Atlantic causes a divergence of wave-activity flux (Methods) to the south of Greenland in the upper troposphere. The wave-activity flux converges toward Euro-Asia before diverging again over the North Pacific, accompanied by anticyclonic and cyclonic anomalies in the “downstream development” (Fig. 6a, b). This pattern of stationary Rossby wave train suggests that the slower AMOC can affect the North Pacific jets through atmospheric teleconnections^32.54^.

Along with the strengthened westerly jets, we also find that the weakened AMOC intensifies storm track activities in the mid-to-high latitudes in the Northern Hemisphere (Fig. 6c). Here, we look into eddy-mean flow interactions using the Eliassen–Palm flux vector containing contributions from both stationary and transient eddies. In response to a weakened AMOC, the Eliassen–Palm flux diverges between 45^o^N and 65^o^N, from surface to about 850 hPa, indicating that changes in eddy flux, primarily those from meridional eddy momentum flux, act to accelerate the low-level westerly winds over the region (Fig. 6d). While the Eliassen–Palm flux converges above and to the south, acting to slow the mean flow there. It merits attention that the Eliassen–Palm flux anomalies should be understood as the positive feedback to, rather than the cause of, the change in the eddy-driven jet.

In the Southern Hemisphere mid-latitudes, westerly jets displace poleward in response to AMOC decline^37,55^ (Fig. 5a), which acts to enhance and diminish the AR frequency to the south and north of 45°S over the Southern Ocean, respectively^20^ (Fig. 4a). The slowed AMOC also generates eastward wind anomalies from the western South Pacific near the Equator to extratropical eastern South Pacific as well as near extratropical South Atlantic (Fig. 5a) to promote ARs in these regions (Fig. 4a).

By contrast, the thermodynamic component, driven by moisture availability, influences global AR frequency, especially those in the mid-to-high latitudes in both hemispheres (Fig. 4b). From 850 hPa specific humidity response to the AMOC decline, we see widespread drying and wetting in the Northern and Southern Hemispheres, respectively, with hemispheric drying stronger than wetting (Fig. 5b). These moisture changes are closely linked to surface air temperature response, with cooling and warming generally across the Northern and Southern Hemispheres, respectively (Fig. 5c). The underlying rationale is that the AMOC slowdown diminishes the northward oceanic heat transport^32^, leading the Northern Hemisphere to cool and the cooler Northern Hemisphere troposphere to contain less water vapor. Likewise, hemispheric characteristics of reduced precipitation north of the equator were previously linked to an AMOC slowdown^56^.

In summary, the weakened AMOC promotes cooling of surface air temperatures and diminished moisture content over the Arctic and Greenland, which lead to a reduction in the thermodynamic component of AR frequency (Figs. 4b, 5b, and 5c). The drying in the Northern Hemisphere contributes to a significant decline in AR frequency over the Arctic and Greenland (Fig. 2c). Such drying effect is minimal in the subtropics, where the dynamic and thermodynamic components of AR frequency counterbalance each other (Figs. 4a, b). In contrast, coastal California experiences an increase in AR activity, driven by the intensified westerly jets over the Pacific, as reflected by changes in westerly winds and the dynamic component of AR frequency (Figs. 4a and 5a). The Southern Hemisphere subtropics, particularly regions of the South Pacific and eastern coast of South America, witness a consistent increase in AR frequency, as mostly driven by dynamic mechanisms but possibly influenced by thermodynamic processes (Figs. 2c, 5b, and 5c). Over the Southern Ocean, AMOC-induced dynamic and thermodynamic components strongly offset each other, with the exception of areas around Antarctica (Fig. 4a, b), resulting in a net response of increased AR frequency over several marginal seas around Antarctica (Fig. 2c).

## Discussions

We leverage fully coupled climate model simulations to investigate the effect of AMOC slowdown on ARs in the twenty-first century. We find that a weakened AMOC increases AR frequency along the western and eastern coasts of North America, and in the western Europe primarily by strengthening Northern Hemisphere westerly winds across the mid-latitude North Pacific and Atlantic Oceans, though this dynamic effect is partially compensated by a thermodynamic effect due to moisture reduction in the Northern Hemisphere. The AMOC-induced hemispheric drying, on the other hand, serves as the main cause of the diminished AR frequency and associated precipitation over the Arctic and Greenland. In the Southern Hemisphere, the decelerated AMOC enhances AR frequency and associated precipitation from the western South Pacific near the Equator to extratropical eastern South Pacific, as well as along the eastern coast of South America, mostly through modifying winds over these regions. The slower AMOC can also increase AR frequency in South, Southeast and East Asia through both dynamic and thermodynamic processes.

In addition to AMOC slowdown and anthropogenic warming, ARs are also influenced by other various teleconnections and modes of natural variability, such as the El Niño–Southern Oscillation^57^ and related Pacific–North American teleconnection^58^, Southern Annular Mode^59^, Pacific Decadal Oscillation and Atlantic Multidecadal Oscillation^60,61^. Although direct comparisons of the response magnitude between natural variability from prior research and our AMOC impact may be difficult due to different AR detection algorithms applied across studies, we perform significance tests on the free- and fixed-AMOC ensembles, presenting where the AMOC-induced AR change is statistically significant relative to that induced by internal climate variability in our simulations (Methods). Furthermore, we illustrate how the AR response to AMOC decline is distinct from responses to other modes. For instance, a positive Pacific Decadal Oscillation is associated with enhanced AR frequency over both the Pacific Arctic sector and California^60,61^, which differs from the pattern of AMOC impact in Fig. 2c.

One may notice that our findings are based on an atmosphere model with a nominal horizontal resolution of one degree, whereas higher horizontal resolutions in atmosphere models^62^ were found to potentially modulate the climatological AR and related precipitation^63,64^. As such, the resolution dependency of the column-integrated precipitable water, vertical profile in the atmosphere, and meridional structure of westerlies may all have an impact on the sensitivity of AR frequency^63^. Therefore, our study calls for exploring the AMOC impact on ARs in high-resolution climate models via a similar free- and fixed-AMOC framework.

## Methods

### Reanalysis data

We examine ARs and the AMOC during the past decades using reanalysis data from NASA MERRA-2 and ECCOv4r4. MERRA-2 starts from 1980, with a nominal horizontal resolution of 0.5^o^×0.625^o^ and includes 42 pressure levels^65^. To detect AR, we calculate IVT by integrating daily mean horizontal wind ($u,v$) and specific humidity ($q$) across four pressure levels: near-surface (1000 hPa), 850 hPa, 500 hPa, and 100 hPa, in the formula,

1 $${IVT}=\,\frac{1}{g}\sqrt{{\left( {\int }_{100}^{1000}{qudp}\right)}^{2}+{\left({\int }_{100}^{1000}{qvdp}\right)}^{2}}.$$

where g represents gravitational acceleration and dp is the pressure difference between consecutive pressure levels. The AR detection algorithm will be detailed in later section. Note that ARs derived from MERRA-2 have been used as a benchmark for evaluating the performance of our climate model in simulating ARs.

ECCOv4r4 is a dynamically and kinematically consistent global ocean reanalysis^66^, having monthly data with a nominal horizontal resolution of ~1° × 1° with 50 vertical levels, spanning from 1992 to 2017. We calculate the Atlantic meridional overturning stream-function ${\psi }_{A}$) at the depth $z$ from the meridional velocity of ocean flow (${v}_{o}$) as,

2 $${\psi }_{A}\left(y,z\right)={\int }_{z}^{0}{\int }_{{x}_{w}}^{{x}_{e}}{v}_{o}\left(x,y,{z}^{{\prime} }\right){dxdz}^{\prime}$$

where $x$, ${y}$ and ${z}^{{\prime} }$ correspond to the zonal, meridional, and vertical depth coordinates, respectively, and ${x}_{w}$ and ${x}_{e}$ represent the western and eastern boundaries of the Atlantic Ocean, respectively. The AMOC strength is defined as the maximum of annual mean meridional stream-function below 500 m in the North Atlantic.

### CESM2 free- and fixed-AMOC simulations

We leverage a broadly used climate model, CESM2, in a configuration of an atmospheric resolution of approximately one degree and a nominal ocean resolution of one degree^44^. We analyze six ensemble members from the CESM2 historical plus SSP5-8.5 simulation (referred to as free-AMOC). In this simulation, the AMOC weakens since the 1980s, as consistent with findings from reanalysis (Fig. 1) and many other climate models^30^.

We also conduct a six-member ensemble sensitivity experiment with CESM2, referred to as fixed-AMOC, in which freshwater is gradually removed from the North Atlantic deep-water formation region^32^ (named RG_FW_, Fig. 1, the inserted map, blue shaded) to maintain the AMOC strength at around 2000 during the twenty-first century (Fig. 1), and evenly redistributed to the rest of global oceans. The fixed-AMOC experiment begins in 2000 and utilizes the same forcing agents as the free-AMOC simulation, following historical conditions prior to 2014 and SSP5-8.5 projections thereafter. As in CESM2 the surface freshwater flux (${F}_{W}$) is represented by a virtual salt flux (${F}_{V}$), that is,

3 $${F}_{V}=-{S}_{{ref}}{F}_{W}$$

where ${S}_{{ref}}=34.7 {psu}$ is the reference salinity. Removing fresh water from the surface of RG_FW_ requires a change of virtual salt flux ($\triangle {F}_{V}$), which is modified as

$$\Delta {F}_{V}=-{S}_{{ref}}\frac{{{FWR}}_{a}}{A}\left(t-2000\right),\,2000 < t\le 2075$$

4 $$\Delta {F}_{V}=-{S}_{{ref}}\frac{{{FWR}}_{a}}{A}\left(t-2000\right)-{S}_{{ref}}\frac{{{FWR}}_{b}}{A}\left(t-2075\right),\,2075 < t\le 2100$$

where $A$ is the area of RG_FW_ in unit of m^2^ and $t$ represents the year index. ${{FWR}}_{a}=8.61\times {10}^{-5}{m}^{3}/{s}^{2}$ and ${{FWR}}_{b}=0.84\times {10}^{-5}{m}^{3}/{s}^{2}$ denote the rates of the change in surface freshwater flux. It merits attention that the freshwater removal area in the North Atlantic is about 2.6% of global ocean area. As a result, the redistributed freshwater to the rest of global oceans is two order smaller in magnitude than the freshwater change in the subpolar North Atlantic, thus imposing neglectable impacts on the climate system.

We focus on two key periods, the historical (1990–2014) and future (2076–2100) periods. We calculate 2076–2100 minus 1990–2014 for climate change and free- minus fixed-AMOC simulation for AMOC impact. Our seasonal analysis is based on December–January–February (DJF) for the boreal winter (austral summer) and June–July–August (JJA) for the boreal summer (austral winter).

### AR detection algorithm

We apply the IVT-based global AR detection algorithm developed by ref.^8^. This approach has been verified through comparison with manual satellite measurements of AR landfall records along the west coast of the United States, spanning latitudes from 34°N to 48°N^67^. The algorithm applies several criteria to identify ARs. While we will briefly summarize some of the key criteria here, a comprehensive description can be found in ref.^8^. In the initial phase of the algorithm, the intensity threshold for identifying contiguous areas (referred to as “objects”) with heightened IVT is determined by the 85th percentile of the IVT magnitude, which varies based on seasonal and regional factors. To maintain the coherence of the object, the algorithm stipulates that more than half of the grid cells within the object must have an average IVT direction that deviates by less than 45 degrees from the overall mean IVT direction of the object. Moreover, the average poleward IVT of the object must exceed 50 kg/m/s. Finally, the object should extend beyond 2,000 kilometers in length, with a length-to-width ratio that exceeds 2. For MERRA-2, CESM2 free- or fixed-AMOC simulation, the 85th percentile of IVT is determined based on the climatology of MERRA-2 or CESM2 free simulation during the historical period of 1990–2014. This threshold is calculated in overlapping five-month time windows, so for any given month and location the threshold will be calculated by using all time steps in the 5-month window that surrounds the current month over the whole length of the dataset. Grid points within each identified AR object are subsequently utilized to calculate AR frequency and AR-induced precipitation.

We examine the probability of AR basic characteristics such as the length and width, lowest latitude and highest latitudes of AR, direction of mean IVT and coherence of IVT direction, mean magnitude of IVT, and landfall magnitude. The lowest and highest latitudes of ARs in either hemisphere measure the meridional range of AR activities. The coherence of the IVT directions with an AR is assessed by the fraction of AR grid cells with IVT directed within 45^o^ of the mean AR IVT^8^.

When we compare AR basic characteristics between MERRA-2 and CESM2 free-AMOC simulation from 1990 to 2014, we find that CESM2 can generally well simulate the AR characteristics, including IVT distribution, AR frequency (Supplementary Fig. 1), seasonal cycle (Supplementary Fig. 7) and landfall latitudes (Supplementary Fig. 8). This result demonstrates CESM2’s fidelity in AR simulation, despite the model’s relatively coarse resolution compared to high-resolution models.

### Dynamic and thermodynamic decomposition of AR frequency

To separate the dynamic and thermodynamic contributions to AR frequency, we scale the specific humidity during the 2076-2100 period relative to its climatological value from the 1990–2014 period. Specifically, the climatological specific humidity (${q}_{c}$) is the time mean from the earlier period, and ${q}_{f}$ denotes the time mean for the later period. The scaling factor (${q}_{c}/{q}_{f}$) is applied to the later period specific humidity, resulting in

5 $${{q}_{{scaled}}=q}_{c}/{q}_{f}\times q$$

where the specific humidity $q$, in particular, represents the unscaled specific humidity at each time step, pressure level, and grid cell. This scaling eliminates the effect of thermodynamic change on AR frequency. The scaled IVT, combined with the IVT threshold from the earlier period, is used to assess the AR response driven by dynamical change, suppressing the effect of background moisture variability. The scaled IVT and the 85th percentile of IVT from the unscaled field is then used as inputs to the AR detection algorithm. The resulting AR frequency from the scaled IVT represents the dynamically driven component, while the difference between total and dynamically driven AR frequencies reflects thermodynamic change^20,68^. The dynamic–thermodynamic decomposition provides a useful first-order diagnostic for interpreting AR responses, but with implicit assumptions of linear separability and fixed circulation. Given the complexity of the interaction between atmospheric temperature gradients and atmospheric circulation, such assumptions may not fully hold for higher-order accuracy. Despite this, dynamic and thermodynamic decompositions successfully capture the key physical drivers of AR changes under different scenarios, such as global warming^69^, Arctic sea ice loss^68^ as well as the AMOC slowdown in this study.

### Eliassen–Palm flux and Takaya–Nakamura and Plumb wave-activity fluxes

We look into eddy-mean flow interactions using the Eliassen–Palm flux vector (${{\bf{F}}}$)^70^,

6 $${{\bf{F}}}=\left(-\overline{u^{\prime} v^{\prime} }\right){{\bf{j}}}+\left(\frac{f\overline{v^{\prime} \theta^{\prime} }}{\overline{{\theta }_{p}}}\right){{\bf{k}}}$$

where overbars and primes denote zonal means and departures therefrom, $f$ is Coriolis parameter, $\theta$ is potential temperature so that $-\bar{{\theta }_{p}}$ measures the static stability at the pressure (*p*) coordinate. ${{\bf{j}}}$ and ${{\bf{k}}}$ denote the unit vectors pointing northward and upward, respectively. The Eliassen–Palm flux vector contains contributions from both stationary and transient eddies, with its vertical component depicting the meridional heat flux and its meridional component depicting the equatorward meridional momentum flux. Using daily data, we calculate the difference of Eliassen–Palm flux vectors for the ensemble mean of CESM2 free- minus fixed-AMOC simulation during 2076–2100.

We also examine the Takaya–Nakamura^71^ and Plumb^72^ wave-activity fluxes for the propagation of AMOC-induced stationary wave. The horizontal components of the Takaya–Nakamura wave-activity flux (${{{\bf{W}}}}_{{{\bf{h}}}}$) are expressed as,

7 $${{{\bf{W}}}}_{{{\bf{h}}}}=\frac{{p}^{*}\cos \phi }{2\left|{{\boldsymbol{U}}}\right|}\left(\begin{array}{l}{\frac{U}{{a}^{2}{\cos }^{2}\phi }\left[{\left(\frac{\partial {\psi }^{{\prime} }}{\partial \lambda }\right)}^{2}-{\psi }^{{\prime} }\frac{{\partial }^{2}{\psi }^{{\prime} }}{\partial {\lambda }^{2}}\right]+\frac{V}{{a}^{2}\cos \phi }\left[\frac{\partial {\psi }^{{\prime} }}{\partial \lambda }\frac{\partial {\psi }^{{\prime} }}{\partial \phi }-{\psi }^{{\prime} }\frac{{\partial }^{2}{\psi }^{{\prime} }}{\partial \lambda \partial \phi }\right]} \\ {\frac{U}{{a}^{2}\cos \phi }\left[\frac{\partial {\psi }^{{\prime} }}{\partial \lambda }\frac{\partial {\psi }^{{\prime} }}{\partial \phi }-{\psi }^{{\prime} }\frac{{\partial }^{2}{\psi }^{{\prime} }}{\partial \lambda \partial \phi }\right]+\frac{V}{{a}^{2}}\left[{\left(\frac{\partial {\psi }^{{\prime} }}{\partial \phi }\right)}^{2}-{\psi }^{{\prime} }\frac{{\partial }^{2}{\psi }^{{\prime} }}{\partial {\phi }^{2}}\right]}\end{array}\right)$$

where ${p}^{*}$ = pressure/1000 hPa, ${\psi }^{{\prime} }$ denotes the perturbation stream-function, ${{\bf{U}}}=\left(U,V,0\right)$ denotes a steady zonally inhomogeneous basic flow with zonal and meridional components of $U$ and $V$, respectively, $a$ is the Earth’s radius, and ($\lambda$, $\phi$) are latitude and longitude, respectively. The horizontal components of the Plumb wave-activity flux (${{{\bf{F}}}}_{{{\bf{sh}}}}$) are expressed as,

8 $${{{\bf{F}}}}_{{{\bf{sh}}}}={pcos}\phi \left(\begin{array}{l}{\frac{U}{{a}^{2}{\cos }^{2}\phi }\left[{\left(\frac{\partial {\psi }^{{\prime} }}{\partial \lambda }\right)}^{2}-{\psi }^{{\prime} }\frac{{\partial }^{2}{\psi }^{{\prime} }}{\partial {\lambda }^{2}}\right]} \\ {\frac{U}{{2a}^{2}\cos \phi }\left[\frac{\partial {\psi }^{{\prime} }}{\partial \lambda }\frac{\partial {\psi }^{{\prime} }}{\partial \phi }-{\psi }^{{\prime} }\frac{{\partial }^{2}{\psi }^{{\prime} }}{\partial \lambda \partial \phi }\right]}\end{array}\right)$$

where the basin flow only includes $U$. Particularly, we examine the annual mean horizontal Takaya–Nakamura and Plumb wave-activity flux anomalies at 250 hPa for the ensemble mean of CESM2 free- minus fixed-AMOC simulation during 2076–2100.

### Significance tests

We primarily show the ensemble mean result of AR change, and meanwhile, realize that internal climate variability can influence ARs. Therefore, we examine the difference between CESM2 free- and fixed-AMOC simulations using the Student’s t-test at the 95% confidence level, which identifies the regions where AMOC impacts are statistically significant relative to internal climate variability. Also, to assess the statistical significance of linear trends, we apply a Student’s t-test and compute the p-value to evaluate whether the trend significantly differs from zero.

## Supplementary information

Supplementary Information

Transparent Peer Review file

## Data Availability

NASA MERRA-2 data are available at (https://gmao.gsfc.nasa.gov/gmao-products/merra-2/data-access_merra-2/). NASA ECCOv4r4 data are available at (https://ecco-group.org/products-ECCO-V4r4.htm). The data and codes to generate Figs. 1–6 are available from Zenodo^73^ at (https://zenodo.org/records/19412480).

## Code availability

Figures are generated via the NCAR Command Language (NCL, Version 6.5.0) [Software]. (2018). Boulder, Colorado: UCAR/NCAR/CISL/TDD (10.5065/D6WD3XH5). The data and codes to generate Figs. 1–6 are available from Zenodo^73^ at (https://zenodo.org/records/19412480).
