# Supplementary material for: Atlantic meridional overturning circulation slowdown modulates atmospheric rivers in a warmer climate
Source: Nat Commun. 2026 May 4;17:5971. doi: 10.1038/s41467-026-72555-w (PMC13342563; doi:10.1038/s41467-026-72555-w)
Supplement: Supplementary file 1 — Supplementary Information [file 41467_2026_72555_MOESM1_ESM.pdf]

Supporting Information

Atlantic meridional overturning circulation slowdown modulates atmospheric rivers in a warmer climate

Mohima Sultana Mimi[1], Wei Liu[1*], Weiming Ma[2], Gang Chen[3]

[1]Department of Earth and Planetary Sciences, University of California Riverside, Riverside, CA USA.
[2]Atmospheric, Climate, and Earth Sciences Division, Pacific Northwest National Laboratory, Richland, WA, USA.
[3]Department of Atmospheric and Oceanic Sciences, University of California Los Angeles, Los Angeles, CA, USA.

*Corresponding author. Email: wei.liu@ucr.edu

Contents of this file
Supplementary Fig. 1 to Fig. 8

Supplementary Figures

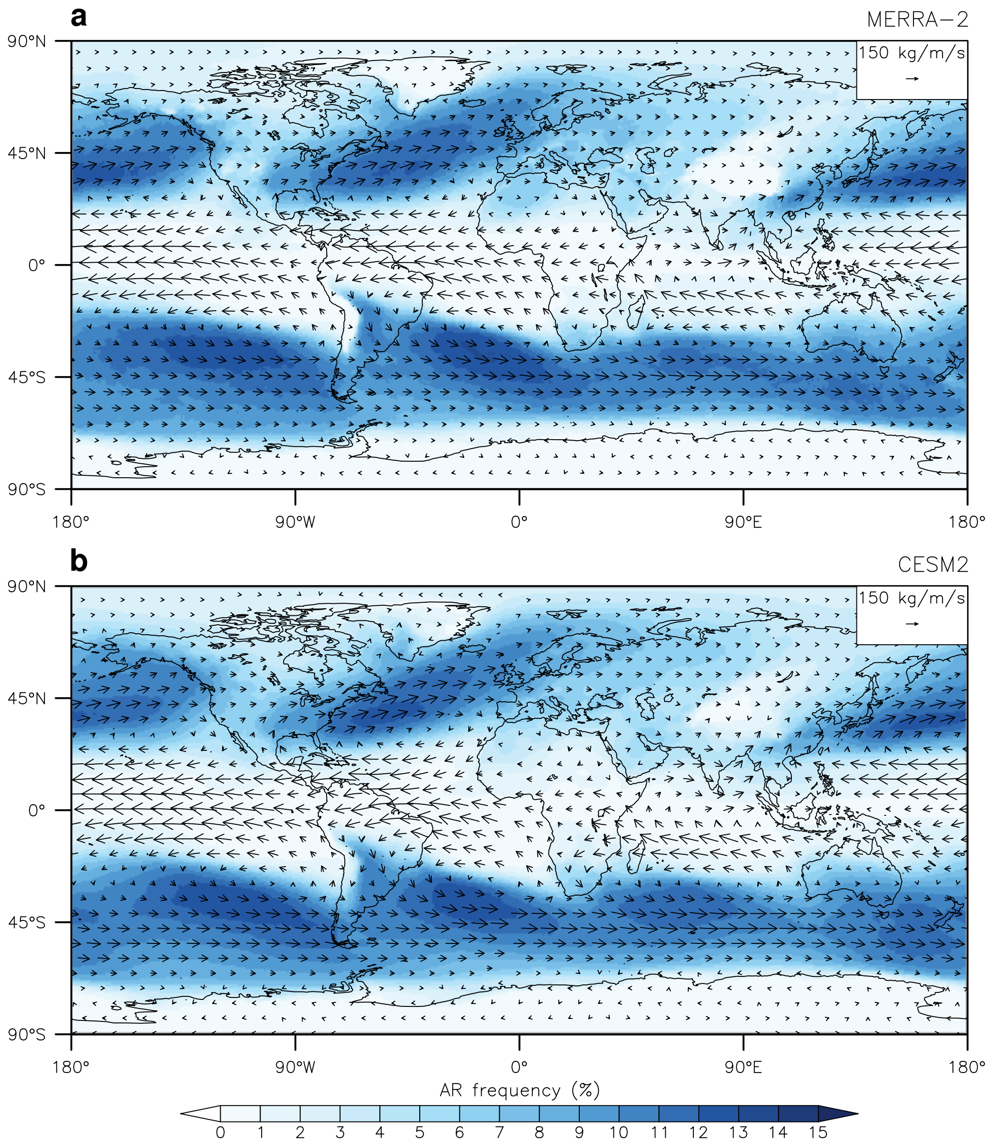

Supplementary Fig. 1. AR frequency and IVT in reanalysis and model simulation. a Annual mean climatological AR frequency (shading in %) and IVT (vectors in kg/m/s) derived from NASA MERRA-2 reanalysis over 1990-2014. **b** Same as **a** but for the ensemble mean of CESM2 free-AMOC simulation over the same period. The base map is from NCAR Command Language map outline databases.

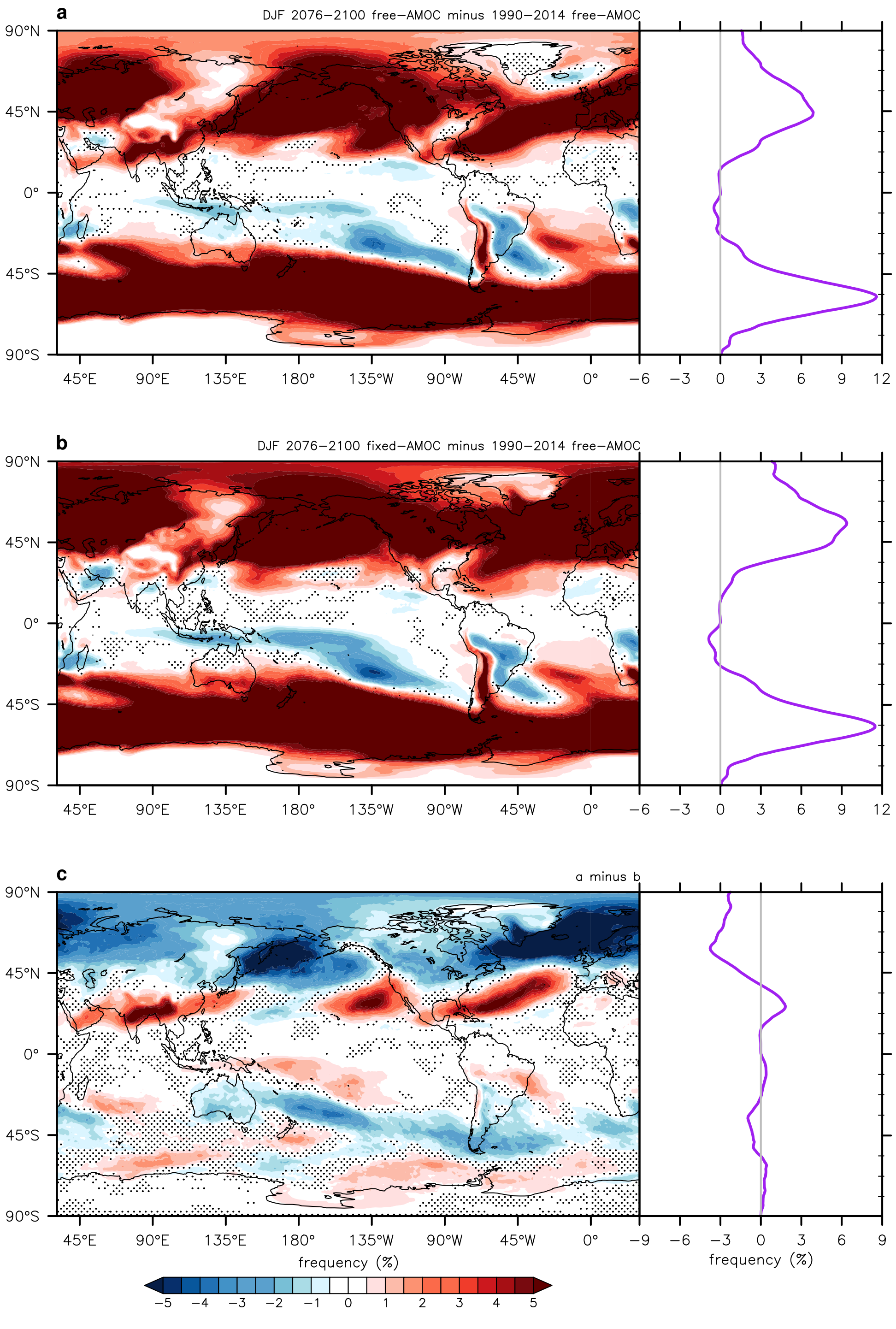

Supplementary Fig. 2. DJF AR frequency change and AMOC impact. Changes in AR frequency (shading in %) for the ensemble means of CESM2 **a** free- and **b** fixed-AMOC simulations during the period of 2076-2100 relative to 1990-2014 during boreal winter (DJF), and **c** the difference between the two (a minus b), along with the zonal mean of AR frequency. The stipples refer to the regions where changes are not significantly different from zero at the 95% confidence level of the Student's t-test. The base map is from NCAR Command Language map outline databases.

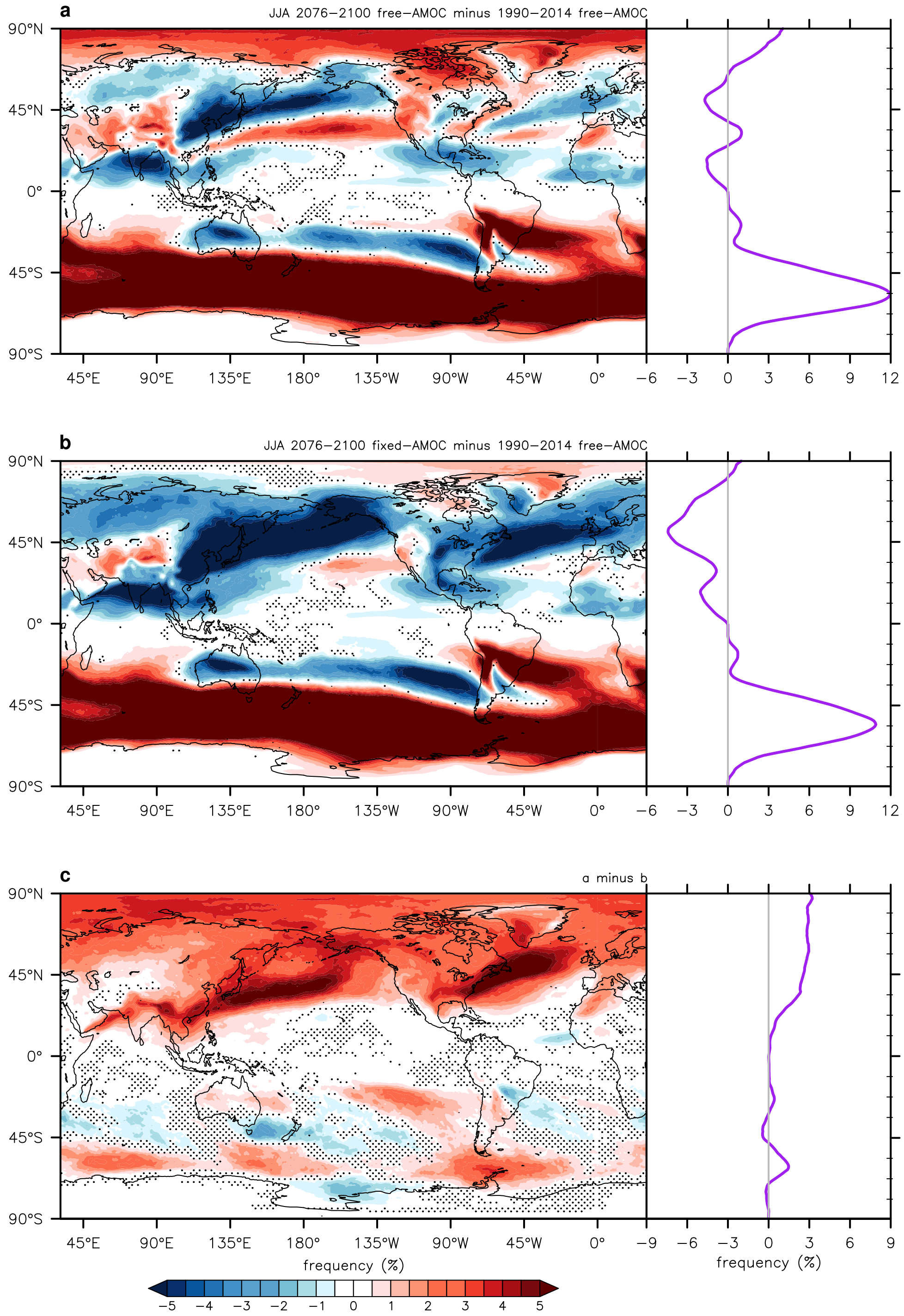

Supplementary Fig. 3. JJA AR frequency change and AMOC impact. Changes in AR frequency (shading in %) for the ensemble means of CESM2 **a** free- and **b** fixed-AMOC simulations during the period of 2076-2100 relative to 1990-2014 during boreal summer (JJA), and **c** the difference between the two (a minus b), along with the zonal mean of AR frequency. The stipples refer to the regions where changes are not significantly different from zero at the 95% confidence level of the Student's t-test. The base map is from NCAR Command Language map outline databases.

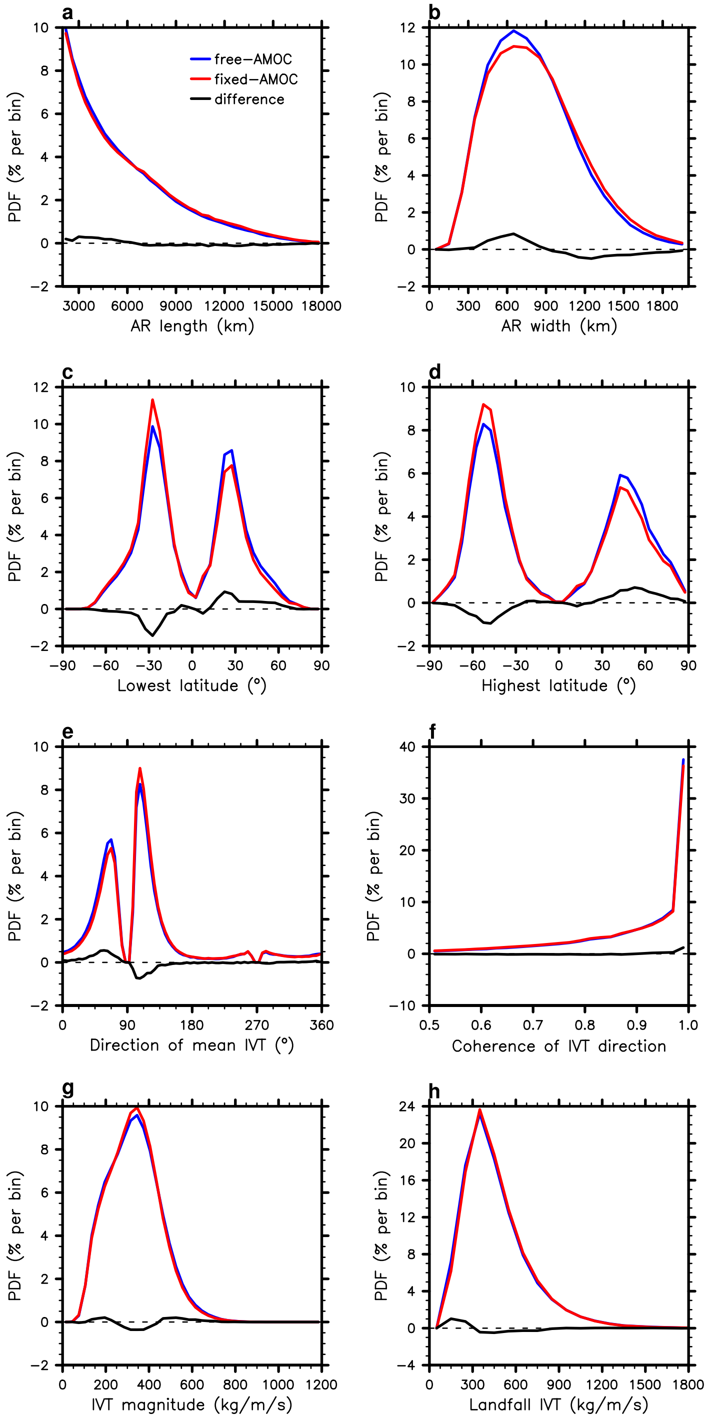

Supplementary Fig. 4. Probability distributions of AR basic characteristics. Probability density functions (PDFs) of the basic characteristics of global ARs for the ensemble means of free- (blue) and fixed-AMOC (red) simulations during 2076-2100, and the difference between the two (free minus fixed, black). **a** length of AR, **b** width of AR, **c** latitude of AR equatorward tip, **d** latitude of AR poleward tip, **e** direction of mean IVT, **f** coherence of IVT direction, **g** magnitude of mean IVT, and **h** magnitude of landfall IVT.

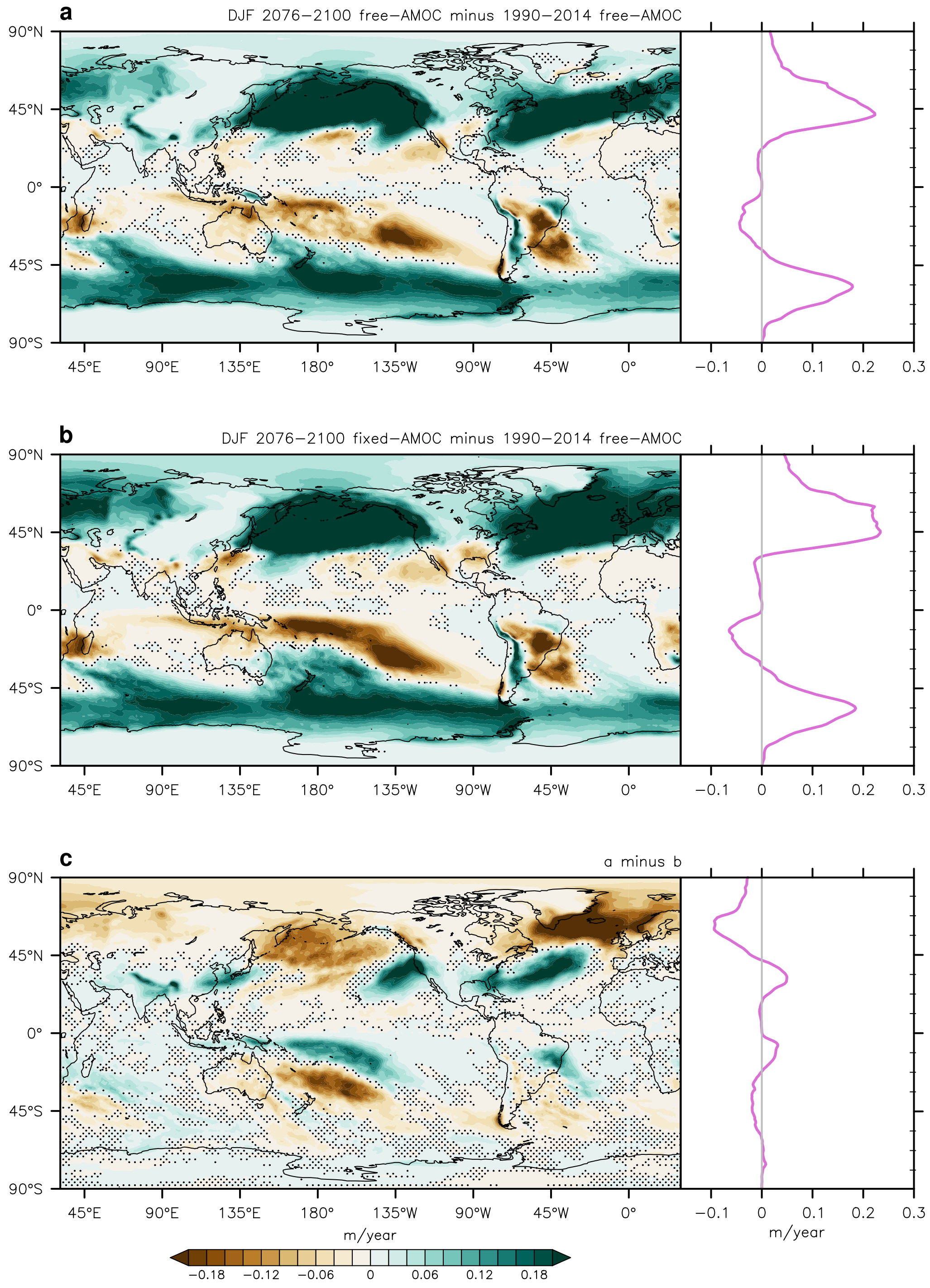

Supplementary Fig. 5. DJF AR-induced precipitation change and AMOC impact. Changes in AR-induced precipitation (shading in m/year) for the ensemble means of CESM2 **a** free- and **b** fixed-AMOC simulations during the period of 2076-2100 relative to 1990-2014 during boreal winter (DJF), and **c** the difference between the two (a minus b), along with the zonal mean of AR-induced precipitation. The stipples refer to the regions where changes are not significantly different from zero at the 95% confidence level of the Student's t-test. The base map is from NCAR Command Language map outline databases.

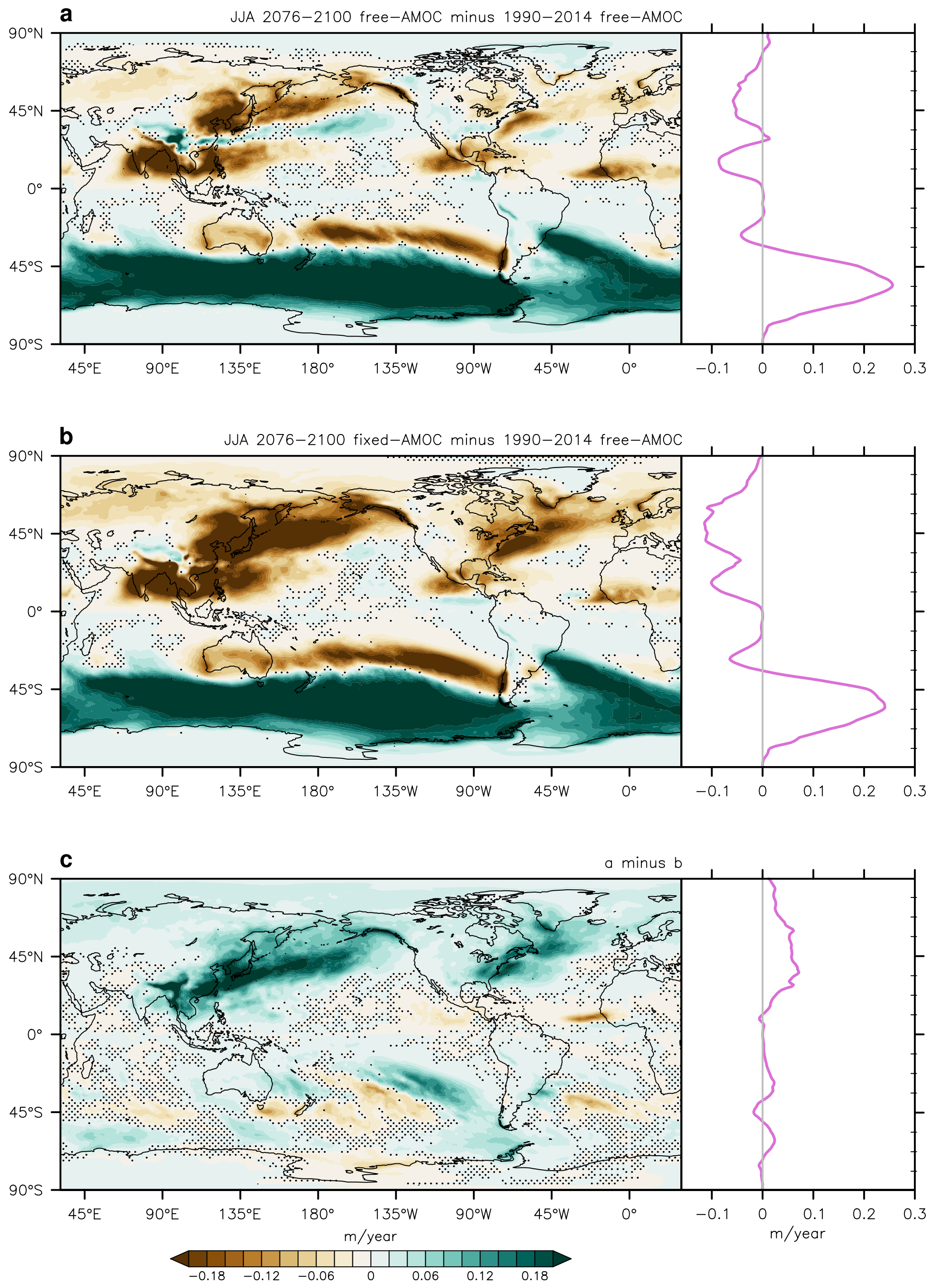

Supplementary Fig. 6. JJA AR-induced precipitation change and AMOC impact. Changes in AR-induced precipitation (shading in m/year) for the ensemble means of CESM2 **a** free- and **b** fixed-AMOC simulations during the period of 2076-2100 relative to 1990-2014 during boreal summer (JJA), and **c** the difference between the two (a minus b), along with the zonal mean of AR-induced precipitation. The stipples refer to the regions where changes are not significantly different from zero at the 95% confidence level of the Student's t-test. The base map is from NCAR Command Language map outline databases.

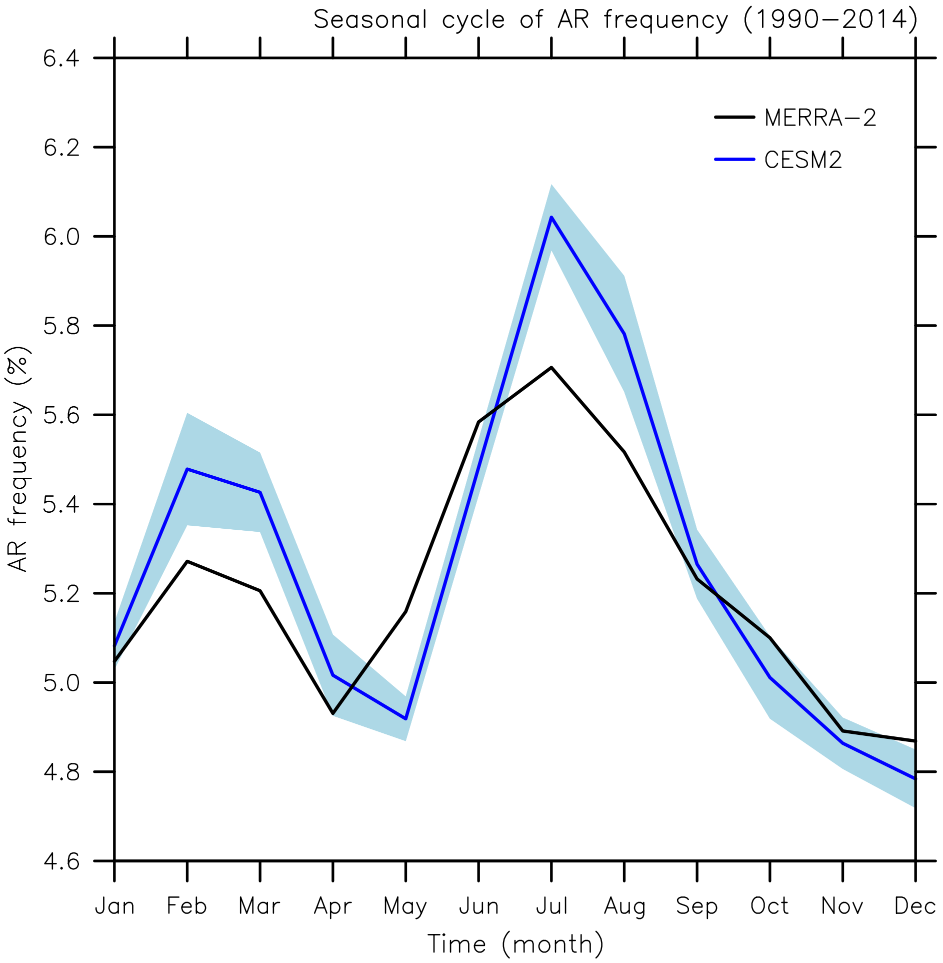

Supplementary Fig. 7. Seasonal cycle of AR frequency in reanalysis and model simulation. Monthly and global mean AR frequencies averaged over 1990–2014 from NASA MERRA-2 reanalysis (black) and CESM2 free-AMOC simulation (ensemble mean, blue; one standard derivation among ensembles, light blue). CESM2 ensemble spread is calculated as one standard deviation among ensembles.

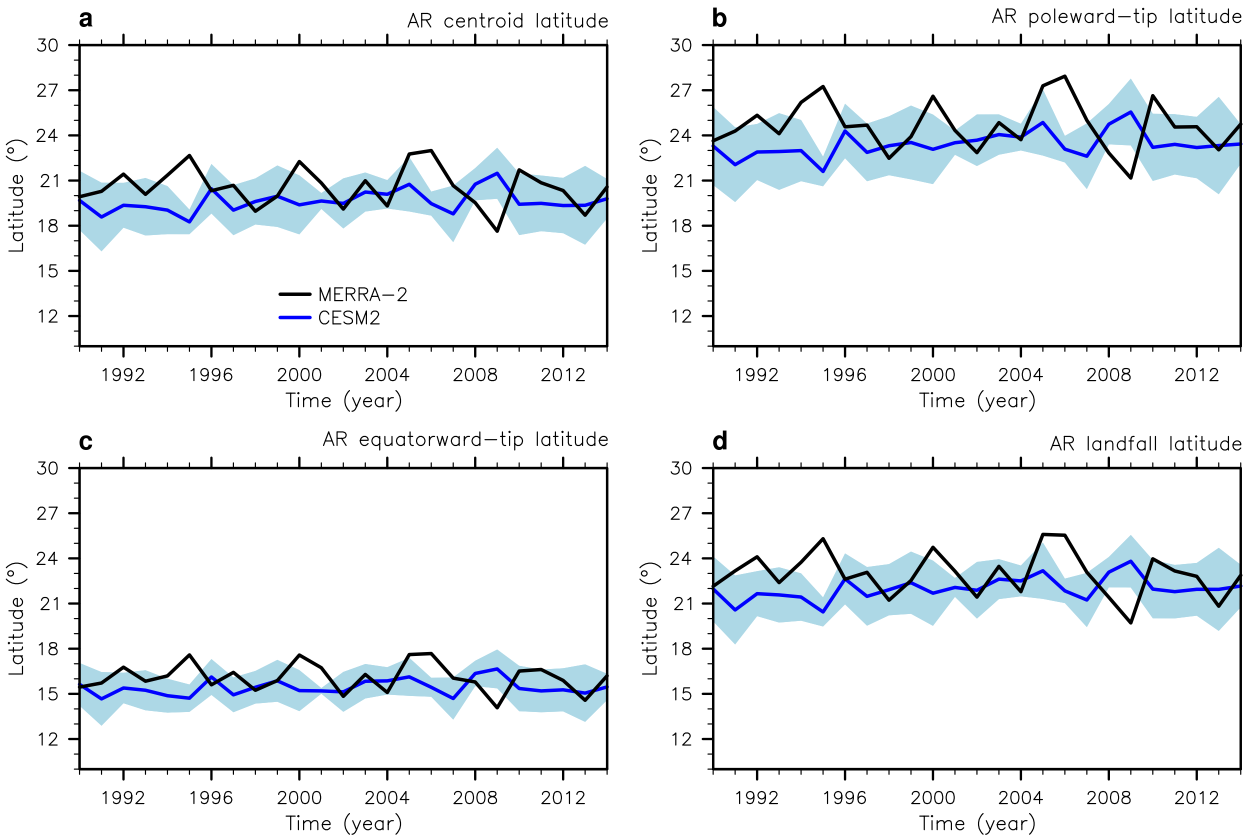

Supplementary Fig. 8. AR latitudes in reanalysis and model simulation. Annual and global mean **a** AR centroid latitude, **b** AR poleward-tip latitude, **c** AR equatorward-tip latitude, and **d** AR landfall latitude over 1990–2014 from NASA MERRA-2 reanalysis (black) and CESM2 free-AMOC simulation (ensemble mean, blue; ensemble spread, light blue). Latitudes are averaged across all detected AR events in each year. CESM2 ensemble spread is calculated as one standard deviation among ensembles.
