# Supplementary material for: Atlantic meridional overturning circulation slowdown modulates atmospheric rivers in a warmer climate
Source: Nat Commun. 2026 May 4;17:5971. doi: 10.1038/s41467-026-72555-w (PMC13342563; doi:10.1038/s41467-026-72555-w)
Supplement: Supplementary file 2 — Transparent Peer Review file [file 41467_2026_72555_MOESM2_ESM.pdf]

nature portfolio

Peer Review File

Atlantic meridional overturning circulation slowdown modulates atmospheric rivers in a warmer climate

Corresponding Author: Dr Wei Liu

This file contains all reviewer reports in order by version, followed by all author rebuttals in order by version.

Attachments originally included by the reviewers as part of their assessment can be found at the end of this file.

Version 0:

Reviewer comments:

Reviewer #1

(Remarks to the Author)
The paper presents research on the impact of the Atlantic meridional overturning circulation (AMOC) on atmospheric rivers (ARs) across the twenty-first century. The work is based on coupled climate model simulations, reanalysis data from MERRA-2 and ECCO v4r4, and aims at relating the weakening of AMOC to enhanced AR frequency in mid-latitudes and a reduction of the AR frequency over the Arctic and Greenland. The subject and the aim of the paper are important - investigating the effect of the AMOC on ARs and associated mechanisms is of great interest because both the AMOC and ARs are subject to alter under climate change, and distinguishing the effect of a weakened AMOC from the influences of other varying and interacting climatic components is not fully understood. The authors found that weakened AMOC enhances AR frequency in mid-latitudes by intensifying the prevailing westerly winds, mainly on the West Coast of North America. The weakening of the AMOC also reduces AR frequency and related precipitation over the Arctic and Greenland. However, the paper unfortunately does not present any significant results that can advance our understanding of this subject. The data analysis, interpretation, and conclusions need additional evidence. For example, the amount of reduction or increase in the frequency of ARs is not quantified. The author found that ARs are most active in the mid-latitudes and less frequent in the tropics and polar regions. The authors used the following terms: ”more frequent”, ”most active”, ”less frequent”, or ”less more frequent” which do not provide any confidence on the results. Can the author provide the percentage increase/decrease in AR associated with 1 million cubic meters per second of AMOC weakening? The authors do not include a discussion section to put their findings in perspective. AMOC and AR are sensitive to global teleconnections, and the authors do not discuss their findings relative to other varying and interacting climate indices. The figures are difficult to read, and the results are presented in a descriptive way, lacking analysis.
Below I raise a number of concerns, including some suggestions for improvement. However, overall the paper needs a new approach/methodology, more in-depth analysis and rewriting.

Please cite the following new study on the MOC decline increases rainfall in parts of the Amazon rainforest.
Hogner, A., Di Capua, G., Donges, J.F., Donner, R.V., Feulner, G., and Wunderling, N. (2025). Causal pathway from AMOC to Southern Amazon rainforest indicates stabilising interaction between two climate tipping elements. Environmental Research Letters DOI: 10.1088/1748-9326/addb62.

1-Line 82. Observed AR means that the authors have performed some fields measurements. Please rephrase, reanalysis are not observation. It should be Reannalysis... Throughout this paragraph, find a different word for observe (lines 83, 88).

2-Line 86-89. Can the author provide some numbers? The Fig. should be accompanied by some numbers in the paragraph. You might add one sentence with the frequency of AR in the mid-latitudes, tropics and the polar region.

3-Line 88-92. You are comparing the MERRA-2 data (1990-2014) and ECCO (1992-2017). Can the authors use the same period for example 1992-2014?

4-Line 90. Please define RAPID.

5-Line 51. Define U.S. West Coast, first use of U.S. Please define.

6-Line 62, the use if this in the ”Mechanisms driving this AR change” seems confusing. I suggest to use ”the” instead.

7-Line 67. Why starting a paragraph with indeed?

8- Line 108. How do the authors know the difference is significative?

9- Line 113-117. The use of ”more frequent”, ”less more frequent” is not precise. The authors should add some numbers. A clear interpretation of the figure is important.

10- Zonal mean profile of what?

11- Line 138. What is the meaning of less frequent here?

12- Line 143, 147. ...the weakened AMOC will cause more frequent ARs in a band extend- ing from the western......... Can the authors indicate the frequency or the number of ARs 13- Line 165. ”Changes are pronounced”. Please add numbers so that the reader can understand without looking at the figures. The figures can not replace the analysis of the results.

14-Line 195. Figure 4c is difficult to read. What is the scale for the wind vector arrow in m/s? I suggest splitting the figures into two pages for visibility. Also please enlarge each panel of Figure 2 for visibility.

15-Line 233-244: Conclusion. Without a discussion section, how does the author know that the increase or decrease in AR frequency is only due to a weakened AMOC? Other climatic indices also modulate the frequency of AR, and the author should find a way to discuss them. The AMOC may be an essential modulator. How important is the AMOC in modulating AR frequency compared to other climatic indices? The author should discuss their finding and examine how important the AMOC modulate AR frequency in comparison to other various teleconnections and modes of natural variability, such as the ENSO,the Southern Annular Mode, the Pacific South American Mode 2, the Pacific Decadal Oscillation Arctic Oscillation, North Atlantic Oscillation, and Pacific/ North America.

Reviewer #2

(Remarks to the Author)
This study addresses an important and timely topic by investigating the linkage between the Atlantic Meridional Overturning Circulation (AMOC) and atmospheric rivers (ARs) under anthropogenic warming. The research presents a novel and innovative perspective, as the direct connection between AMOC variability and AR behavior has rarely been explored in previous studies. The results are potentially significant and may provide new insights into how large-scale ocean circulation can modulate regional hydroclimate extremes.However, the current version of the manuscript still requires substantial improvement in the explanation of physical mechanisms and experiment design to strengthen the robustness and credibility of the conclusions. Therefore, I recommend Major Revision before further publication.

Summary
This manuscript investigates how a weakening AMOC under anthropogenic warming influences AR behavior throughout the twenty-first century. Using reanalysis data and coupled climate model simulations with both free-AMOC and fixed-AMOC configurations in CESM2, the authors aim to isolate the direct impact of AMOC slowdown from other concurrent climate forcings. The study finds that a weakened AMOC enhances AR frequency and AR-induced precipitation in the Northern Hemisphere mid-latitudes—most notably along the U.S. West Coast—through intensified westerly winds, while simultaneously reducing AR activity and related precipitation over the Arctic and Greenland due to reduced moisture availability. In contrast, AR frequency increases along the eastern coast of South America and parts of the South Pacific, associated with AMOC-induced moistening in the Southern Hemisphere. The authors attribute these responses to two main mechanisms: (1) dynamic effects linked to circulation and jet-stream changes, and (2) thermodynamic effects related to hemispheric moisture redistribution. Overall, the work highlights the potential role of AMOC variability in shaping future regional hydroclimate and extreme precipitation patterns.

Major Comments
(1) Incomplete motivation for connecting AMOC and ARs
Line 71–73: When introducing the potential linkage between the AMOC and atmospheric rivers, the authors are encouraged to provide more explicit reasoning and supporting references rather than relying solely on the brief statement “The ongoing and projected declines of the AMOC could profoundly affect various elements in the global climate system, encompassing ARs.” This phrasing is too general. The motivation would be strengthened by clarifying why and how a weakened AMOC could influence ARs—for example, through changes in sea-surface-temperature patterns, shifts in the westerly jet, or alterations in large-scale moisture transport—supported by relevant literature.

(2) Design of CESM2 experiments

Line 99–109: Please provide a table summarizing the design of the CESM2 experiments, which would help readers better identify the experimental differences. I have some concerns regarding the fixed-AMOC configuration. The fixed-AMOC setup artificially removes freshwater from the North Atlantic and redistributes it globally to maintain overturning strength. While this is a common technique in AMOC sensitivity studies, it inevitably alters surface density and SST patterns beyond the AMOC region, potentially introducing spurious atmospheric responses. The authors should discuss these side effects and clarify how they isolate the AMOC signal from the freshwater perturbation itself.
Although the fixed-AMOC experiment maintains the overturning streamfunction, it may not fully constrain the associated SST or salinity gradients. Thus, part of the atmospheric response attributed to AMOC weakening might arise from residual oceanic adjustments rather than the overturning strength per se. Clarifying this distinction would improve the physical interpretation.
Before interpreting the AR frequency difference as a pure AMOC signal, the authors should verify that the associated SST, SLP, and IVT anomalies between the free- and fixed-AMOC simulations are physically consistent with known AMOC teleconnection patterns.

(3) Statement about AMOC weakening and increased AR frequency
Line 121–127 and Fig. 2: I still have some concerns about how a weakened AMOC increases AR frequency. Please demonstrate more clearly the causal chain “AMOC slowdown → circulation change → increased AR activity.”
For the North Atlantic and other mid-latitude regions, the underlying mechanisms may differ. In the North Atlantic, local air–sea interactions and SST gradients may dominate, whereas in other regions teleconnections or jet-dynamics changes in the westerlies could play a larger role.
Additionally, could the authors show global AR frequency changes based on observations or reanalysis data for validation?

(4) Decomposition technique
The dynamic/thermodynamic decomposition is a useful first-order diagnostic, but it implicitly assumes linear separability and fixed circulation. Given that AMOC-induced temperature gradients can alter baroclinicity and atmospheric stability, this assumption may not hold. Please explain the potential limitations of this approach and how they affect your results.

(5) Physical mechanisms
Line 189: The manuscript attributes westerly-jet intensification to transient-eddy forcing and global teleconnections without providing diagnostics to support this claim. Including eddy momentum flux, E–P flux divergence, or storm-track activity maps would substantially strengthen the physical interpretation and demonstrate the mechanism driving AR changes.
In addition, a schematic or conceptual cartoon summarizing the proposed mechanisms would be a valuable addition to the paper.

Minor Comments
Line 94: Please include the citation for CESM2. Is there any specific reason for choosing this model?
Figure 1: The figure appears to be of low quality; details are difficult to discern in the PDF version.
Line 114: “less more frequent” should be corrected to “less frequent.”

Overall Recommendation
The manuscript presents a potentially valuable contribution to understanding AMOC–AR interactions under global warming. However, the physical reasoning and methodological transparency require further strengthening before publication. Addressing the above points—especially the experiment design justification and the diagnostic evidence for dynamic mechanisms—would significantly enhance the paper’s scientific rigor and clarity.

Reviewer #3

(Remarks to the Author)
The manuscript needs substantial revision. Below are my comments

Major comments:

1. The manuscript relies heavily on the contrast between free-AMOC and fixed-AMOC simulations. However, the methodological description of the freshwater extraction strategy (page 10–11) lacks quantitative details:
How much freshwater is removed annually?How sensitive is the AMOC stabilization to spatial pattern and magnitude of removal?

2. Could unintended circulation/stratification artifacts occur in the subpolar gyre?

3. A clearer justification and a sensitivity demonstration are essential, since the entire attribution of AMOC impacts depends on this experimental setup.

4. Although MERRA-2 ARs are used as a benchmark, the manuscript does not present a systematic evaluation of CESM2’s fidelity in AR characteristics (frequency, IVT distribution, seasonal cycle, landfall latitudes). Given known issues in coarse-resolution AR representation (especially jet latitude biases, storm-track spread), readers need at least one figure/table comparing CESM2 vs. MERRA-2 or other ARTMIP datasets. This is crucial before inferring robust future changes.

5. The dynamic response figure (page 22) shows a strong intensification of Pacific westerlies under AMOC slowdown.

However, the mechanisms remain vaguely described.Is this a teleconnection via stationary waves (as suggested in Liu et al. 2020)?

How do SST anomalies propagate from the North Atlantic into the Pacific jet region ? What is the relative contribution of transient eddies vs. mean-flow adjustment?
A more mechanistic, process-level explanation—possibly including zonal-mean wind anomalies or wave activity flux—would significantly strengthen the paper.

Minor comments:

1. The 85th percentile IVT threshold is recomputed separately for 1990–2014 and 2076–2100 (page 10). This may artificially shift AR frequency due to changing climatological moisture. Authors should comment on how this choice affects comparability.

2. Some figures (e.g., dynamic/thermodynamic responses on page 22) lack clear unit notation in captions. Units for frequency change (%) vs. absolute frequency need explicit distinction.

3. The zonal means on the right panels (pages 20–21) are difficult to interpret due to compressed axis scaling. Consider rescaling axes or enlarging the side panels for clarity.

Version 1:

Reviewer comments:

Reviewer #1

[Editorial Note: Please also see attachment at end of file]

(Remarks to the Author)
The current version of the manuscript provides significant analyses on the impact of the Atlantic meridional overturning circulation (AMOC) on atmospheric rivers (ARs) throughout the twenty-first century. The authors have answered most of my concerns.

1.1 Major Comments
With minor revisions, the manuscript could improve our understanding of the interactions between the AMOC and the AR in a warming climate. The authors found that weakened AMOC increases AR frequency in mid-latitudes by intensifying the prevailing westerly winds, particularly along the West Coast of North America.
• What is still missing is how an increase in AR frequency impacts the West Coast of North America, for example. Corringham et al. suggested that ARs are responsible for the majority of economic losses from flooding in the Western United States and are expected to intensify in a warming climate. They argued that flood damage from extreme AR events may triple, from 1 billion to over 3 billion a year, toward the end of the century if mitigation actions are not implemented to reduce anthropogenic greenhouse gas emissions.
Can the author examine or discuss how he by 0.18 m/year (up to 0.44 m/year) enhance AR-induced precipitation of 0.18 m/year (up to 0.44 m/year) caused by the weakened AMOC will affect the US West Coast?
Corringham TW, McCarthy J, Shulgina T, Gershunov A, Cayan DR, Ralph FM. Cli- mate change contributions to future atmospheric river flood damages in the western United States. Nature Scientific Reports. 2022;12:13747. DOI: 10.1038/s41598-022- 15474-2
• The section about the Eliassen-Palm flux and the Takaya-Nakamura and Plumb wave- activity fluxes needs more details. The section about the Eliassen-Palm flux and the Takaya-Nakamura and Plumb wave-activity fluxes needs more details. In equations 6 (line 413), 7 (line 426), and 8 (line 431), how the perturbation stream-function or the departures from the zonal means were computed. Did the authors use a turbulence closure scheme?

Reviewer #2

(Remarks to the Author)
Thank you for the thorough revision and detailed responses. The authors have adequately addressed the previous concerns and strengthened the manuscript with additional analyses and improved clarity. I recommend acceptance in its current form.

Reviewer #3

(Remarks to the Author)
I am satisfied with the review and recommend acceptance of the manuscript.

Open Access This Peer Review File is licensed under a Creative Commons Attribution 4.0 International License, which permits use, sharing, adaptation, distribution and reproduction in any medium or format, as long as you give appropriate credit to the original author(s) and the source, provide a link to the Creative Commons license, and indicate if changes were made.
In cases where reviewers are anonymous, credit should be given to 'Anonymous Referee' and the source.
The images or other third party material in this Peer Review File are included in the article's Creative Commons license, unless indicated otherwise in a credit line to the material. If material is not included in the article's Creative Commons license and your intended use is not permitted by statutory regulation or exceeds the permitted use, you will need to obtain permission directly from the copyright holder.
To view a copy of this license, visit https://creativecommons.org/licenses/by/4.0/

Reviewer #1 (Remarks to the Author):

The paper presents research on the impact of the Atlantic meridional overturning circulation (AMOC) on atmospheric rivers (ARs) across the twenty-first century. The work is based on coupled climate model simulations, reanalysis data from MERRA-2 and ECCO v4r4, and aims at relating the weakening of AMOC to enhanced AR frequency in mid-latitudes and a reduction of the AR frequency over the Arctic and Greenland. The subject and the aim of the paper are important - investigating the effect of the AMOC on ARs and associated mechanisms is of great interest because both the AMOC and ARs are subject to alter under climate change, and distinguishing the effect of a weakened AMOC from the influences of other varying and interacting climatic components is not fully understood. The authors found that weakened AMOC enhances AR frequency in mid-latitudes by intensifying the prevailing westerly winds, mainly on the West Coast of North America. The weakening of the AMOC also reduces AR frequency and related precipitation over the Arctic and Greenland.

However, the paper unfortunately does not present any significant results that can advance our understanding of this subject. The data analysis, interpretation, and conclusions need additional evidence. For example, the amount of reduction or increase in the frequency of ARs is not quantified. The author found that ARs are most active in the mid-latitudes and less frequent in the tropics and polar regions. The authors used the following terms: ”more frequent”, ”most active”, ”less frequent”, or ”less more frequent” which do not provide any confidence on the results. Can the author provide the percentage increase/decrease in AR associated with 1 million cubic meters per second of AMOC weakening? The authors do not include a discussion section to put their findings in perspective. AMOC and AR are sensitive to global teleconnections, and the authors do not discuss their findings relative to other varying and interacting climate indices. The figures are difficult to read, and the results are presented in a descriptive way, lacking analysis.

Below I raise a number of concerns, including some suggestions for improvement. However, overall the paper needs a new approach/methodology, more in-depth analysis and rewriting.

Thanks for the insightful advice and comments!

Following reviewer’s comment, we use a common historical (1990-2014) climatology from CESM2 free simulation to analyze the 85th percentile of IVT and ARs in all the simulations for all the time periods. Thus, we amend Figs. 2, 3 and 4, as well as corresponding supplementary figures, but our main finding remains unchanged, indicating that the choice of baseline climatology to compute IVT and AR has little effect on our conclusions of AMOC impacts on ARs and AR-induced precipitation.

Please cite the following new study on the MOC decline increases rainfall in parts of the Amazon rainforest.
Hogner, A., Di Capua, G., Donges, J.F., Donner, R.V., Feulner, G., and Wunderling, N. (2025). Causal pathway from AMOC to Southern Amazon rainforest indicates stabilising interaction between two climate tipping elements. Environmental Research Letters DOI: 10.1088/1748-9326/addb62.
Cited as suggested.

1-Line 82. Observed AR means that the authors have performed some fields measurements. Please rephrase, reanalysis are not observation. It should be Reanalysis… Throughout this paragraph, find a different word for observe (lines 83, 88).

Revised as suggested.

2-Line 86-89. Can the author provide some numbers? The Fig. should be accompanied by some numbers in the paragraph. You might add one sentence with the frequency of AR in the mid-latitudes, tropics and the polar region.
Yes, we find that during 1992-2014, the annual mean AR frequency is 9.04% in the mid-latitudes, which is substantially higher than those in the tropics (2.74%) and polar (3.89%) regions, respectively. We have added the above information to the revision.

3-Line 88-92. You are comparing the MERRA-2 data (1990-2014) and ECCO (1992-2017). Can the authors use the same period for example 1992-2014?
Sure. We use the same period of 1992-2014 in the revision.

4-Line 90. Please define RAPID.
Revised as suggested, i.e., RAPID is defined in the second paragraph where it is firstly used.

5-Line 51. Define U.S. West Coast, first use of U.S. Please define.
Revised as suggested.

6-Line 62, the use if this in the" Mechanisms driving this AR change" seems confusing. I suggest to use" the" instead.
Revised as suggested.

7-Line 67. Why starting a paragraph with indeed?
"Indeed" has been removed.

8- Line 108. How do the authors know the difference is significative?
As clarified in the section of "Significance tests", We examine the differences between CESM2 free- and fixed-AMOC simulations using the Student's t-test at the 95% confidence level. This analysis highlights the regions where AMOC impacts are statistically significant relative to internal climate variability. The related results were included in Figs. 2-5 as well as associated supplementary figures.

9- Line 113-117. The use of "more frequent", "less more frequent" is not precise. The authors should add some numbers. A clear interpretation of the figure is important.
Sure. We have added the numbers to the revision for a clear interpretation.

10- Zonal mean profile of what?
Zonal mean profile of AR frequency. We clarify it in the revision.

11- Line 138. What is the meaning of less frequent here?
Less frequent ARs. It means less frequent ARs over the Gulf region in the fixed AMOC case than the weakened AMOC case.

12- Line 143, 147. ...the weakened AMOC will cause more frequent ARs in a band extending from the western......... Can the authors indicate the frequency or the number of ARs
Yes. We have added the numbers to this paragraph.

13- Line 165. ”Changes are pronounced”. Please add numbers so that the reader can understand without looking at the figures. The figures can not replace the analysis of the results.
Following the review, we have added the numbers to this paragraph.

14-Line 195. Figure 4c is difficult to read. What is the scale for the wind vector arrow in m/s? I suggest splitting the figures into two pages for visibility. Also please enlarge each panel of Figure 2 for visibility.
Fig. 4c shows changes in 850 hPa zonal wind (shading in m/s) where the contours show the climatology of the zonal wind. There is no wind vector arrow in the figure. Following the review, we have split Fig. 4 into two figures in the revision and enlarged the panels in Fig. 2 for visibility. We have also changed the figure format to “eps” for better visibility.

15-Line 233-244: Conclusion. Without a discussion section, how does the author know that the increase or decrease in AR frequency is only due to a weakened AMOC? Other climatic indices also modulate the frequency of AR, and the author should find a way to discuss them. The AMOC may be an essential modulator. How important is the AMOC in modulating AR frequency compared to other climatic indices? The author should discuss their finding and examine how important the AMOC modulate AR frequency in comparison to other various teleconnections and modes of natural variability, such as the ENSO, the Southern Annular Mode, the Pacific South American Mode 2, the Pacific Decadal Oscillation Arctic Oscillation, North Atlantic Oscillation, and Pacific/North America.
Yes, we agree with the reviewer. In the paper, we show the ensemble mean result of AR change, and meanwhile, realize that internal climate variability such as the El Niño–Southern Oscillation (Kim et al. 2019) and related Pacific–North American teleconnection (Luna-Niño et al. 2025), Southern Annular Mode (Baek et al. 2023), Pacific Decadal Oscillation and Atlantic Multidecadal Oscillation (Ma et al. 2024; Dong et al. 2021) can also influence ARs. Although direct comparisons of the response magnitude between natural variability from prior research and our AMOC impact may be difficult due to different AR detection algorithms applied across studies, we perform significance tests on the free- and fixed-AMOC ensembles, presenting where the AMOC-induced AR change is statistically significant relative to that induced by internal climate variability in our simulations. In particular, we examine the differences between CESM2 free- and fixed-AMOC simulations using the Student’s t-test at the 95% confidence level, which identifies the regions where AMOC impacts are statistically significant relative to internal climate variability. We have clarified this in the section of “Significance tests”.

Furthermore, we illustrate how the AR response to AMOC decline is distinct from responses to other modes. For instance, a positive Pacific Decadal Oscillation is associated with enhanced AR frequency over both the Pacific Arctic sector and California, which differs from the pattern of AMOC impact in Fig. 2c.

We have added the above discussion in the section of “Conclusion and discussions” in the revision.

Baek, S.H., Battalio, J.M. and Lora, J.M., 2023. Atmospheric river variability over the last millennium driven by annular modes. AGU Advances, 4, e2022AV000834.

Dong, L., Leung, L.R., Song, F. and Lu, J., 2021. Uncertainty in El Niño-like warming and California precipitation changes linked by the Interdecadal Pacific Oscillation. Nature Communications, 12, 6484.

Kim, H.M., Zhou, Y. and Alexander, M.A., 2019. Changes in atmospheric rivers and moisture transport over the Northeast Pacific and western North America in response to ENSO diversity. Climate Dynamics, 52, 7375-7388.

Luna-Niño, R., Gershunov, A., Ralph, F. M., Weyant, A., Guirguis, K., DeFlorio, M. J., et al. (2025). Heresy in ENSO teleconnections: atmospheric rivers as disruptors of canonical seasonal precipitation anomalies in the Southwestern US. Climate Dynamics, 63, 115.

Ma, W., Wang, H., Chen, G., Leung, L. R., Lu, J., Rasch, P. J., et al. (2024). The role of interdecadal climate oscillations in driving Arctic atmospheric river trends. Nature Communications, 15, 2135.

Reviewer #2 (Remarks to the Author):

This study addresses an important and timely topic by investigating the linkage between the Atlantic Meridional Overturning Circulation (AMOC) and atmospheric rivers (ARs) under anthropogenic warming. The research presents a novel and innovative perspective, as the direct connection between AMOC variability and AR behavior has rarely been explored in previous studies. The results are potentially significant and may provide new insights into how large-scale ocean circulation can modulate regional hydroclimate extremes. However, the current version of the manuscript still requires substantial improvement in the explanation of physical mechanisms and experiment design to strengthen the robustness and credibility of the conclusions. Therefore, I recommend Major Revision before further publication.

Summary
This manuscript investigates how a weakening AMOC under anthropogenic warming influences AR behavior throughout the twenty-first century. Using reanalysis data and coupled climate model simulations with both free-AMOC and fixed-AMOC configurations in CESM2, the authors aim to isolate the direct impact of AMOC slowdown from other concurrent climate forcings. The study finds that a weakened AMOC enhances AR frequency and AR-induced precipitation in the Northern Hemisphere mid-latitudes—most notably along the U.S. West Coast—through intensified westerly winds, while simultaneously reducing AR activity and related precipitation over the Arctic and Greenland due to reduced moisture availability. In contrast, AR frequency increases along the eastern coast of South America and parts of the South Pacific, associated with AMOC-induced moistening in the Southern Hemisphere. The authors attribute these responses to two main mechanisms: (1) dynamic effects linked to circulation and jet-stream changes, and (2) thermodynamic effects related to hemispheric moisture redistribution. Overall, the work highlights the potential role of AMOC variability in shaping future regional hydroclimate and extreme precipitation patterns.

Thanks for the insightful advice and comments!

Following reviewer's comment, we use a common historical (1990-2014) climatology from CESM2 free simulation to analyze the 85th percentile of IVT and ARs in all the simulations for all the time periods. Thus, we amend Figs. 2, 3 and 4, as well as corresponding supplementary figures, but our main finding remains unchanged, indicating that the choice of baseline climatology to compute IVT and AR has little effect on our conclusions of AMOC impacts on ARs and AR-induced precipitation.

Major Comments
(1) Incomplete motivation for connecting AMOC and ARs
Line 71–73: When introducing the potential linkage between the AMOC and atmospheric rivers, the authors are encouraged to provide more explicit reasoning and supporting references rather than relying solely on the brief statement "The ongoing and projected declines of the AMOC could profoundly affect various elements in the global climate system, encompassing ARs." This phrasing is too general. The motivation would be strengthened by clarifying why and how a weakened AMOC could influence ARs—for example, through changes in sea-surface-temperature patterns, shifts in the westerly jet, or alterations in large-scale moisture transport—supported by relevant literature.
Following the review, we have enriched the motivation as "The ongoing and projected declines of the AMOC could profoundly affect the global climate system—for example, modifying the pattern

of sea-surface temperature change (Vellinga and Wood 2008; Liu et al. 2020) and displacing the tropical rain belt southward (Zhang and Delworth 2005), modulating North Atlantic jets, storm tracks and moisture transports (Woollings et al. 2012; Hand et al. 2019; Liu et al. 2020), all of which will potentially influence ARs."

Hand, R., Keenlyside, N.S., Omrani, N.E., Bader, J. and Greatbatch, R.J., 2019. The role of local sea surface temperature pattern changes in shaping climate change in the North Atlantic sector. Climate Dynamics, 52, 417-438.

Hogner, A., Di Capua, G., Donges, J.F., Donner, R.V., Feulner, G., and Wunderling, N., 2025. Causal pathway from AMOC to Southern Amazon rainforest indicates stabilising interaction between two climate tipping elements. Environmental Research Letters, 20, 074026.

Liu, W., Fedorov, A. V., Xie, S. P., and Hu, S., 2020. Climate impacts of a weakened Atlantic Meridional Overturning Circulation in a warming climate. Science Advances, 6, eaaz4876.

Vellinga, M. and Wood, R.A., 2008. Impacts of thermohaline circulation shutdown in the twenty-first century. Climatic Change, 91, 43-63.

Woollings, T., Gregory, J.M., Pinto, J.G., Reyers, M. and Brayshaw, D.J., 2012. Response of the North Atlantic storm track to climate change shaped by ocean–atmosphere coupling. Nature Geoscience, 5, 313-317.

Zhang, R. and Delworth, T.L., 2005. Simulated tropical response to a substantial weakening of the Atlantic thermohaline circulation. Journal of climate, 18, 1853-1860.

(2) Design of CESM2 experiments
Line 99–109: Please provide a table summarizing the design of the CESM2 experiments, which would help readers better identify the experimental differences. I have some concerns regarding the fixed-AMOC configuration. The fixed-AMOC setup artificially removes freshwater from the North Atlantic and redistributes it globally to maintain overturning strength. While this is a common technique in AMOC sensitivity studies, it inevitably alters surface density and SST patterns beyond the AMOC region, potentially introducing spurious atmospheric responses. The authors should discuss these side effects and clarify how they isolate the AMOC signal from the freshwater perturbation itself.
In response to review, we have added more information about the fixed-AMOC configuration in the revision. The freshwater removal area in the North Atlantic is about 9.5×10^{12} m^2, account for about 2.6% of global ocean area. As a result, the redistributed freshwater to the rest of global oceans is two order smaller in magnitude than the freshwater forcing in the subpolar North Atlantic, which will impose neglectable impact in the climate system.

Although the fixed-AMOC experiment maintains the overturning streamfunction, it may not fully constrain the associated SST or salinity gradients. Thus, part of the atmospheric response attributed to AMOC weakening might arise from residual oceanic adjustments rather than the overturning strength per se. Clarifying this distinction would improve the physical interpretation.
Yes, in the fixed-AMOC experiments, SST will still change in response to anthropogenic warming, so does SST gradient. Thus, the difference between the free- and fixed-AMOC simulations allows the cancellation of the part of SST change due to anthropogenic warming so as to reflect the part of

SST change due to AMOC weakening. In a coupled climate system, the AMOC weakening will bring about oceanic adjustments, for example, to decelerate the subpolar gyre circulation in the North Atlantic, which, involves atmospheric response and ocean-atmosphere interaction in that region (Mimi and Liu 2024). But these associated oceanic and atmospheric changes are all of part of the effects of AMOC weakening, benefited from our experimental setup in a coupled system, and included in the difference between the free- and fixed-AMOC simulations.

Mimi, M. S., and Liu, W., 2024. Atlantic Meridional Overturning Circulation slowdown modulates wind-driven circulations in a warmer climate. Communications Earth & Environment, 5, 727.

Before interpreting the AR frequency difference as a pure AMOC signal, the authors should verify that the associated SST, SLP, and IVT anomalies between the free- and fixed-AMOC simulations are physically consistent with known AMOC teleconnection patterns.

The SST difference between our CESM2 free- and fixed-AMOC simulations (Fig. R1) is consistent with surface air temperature change (Fig. 4f in the original submission), both of which illustrate a well-known bipolar seesaw pattern—cooling in the Northern Hemisphere but warming in the Southern Hemisphere—in response to a weakened AMOC as reported in many previous models (e.g., Stouffer et al. 2006).

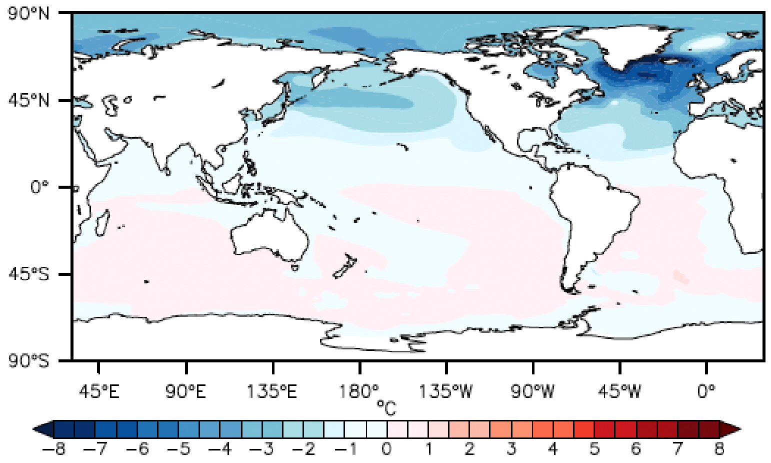

Fig. R1. AMOC impact of global sea surface temperature (SST). Annual mean SST difference (shading in °C) between the ensemble means of CESM2 free- and fixed-AMOC simulations during the period of 2076-2100 (free minus fixed).

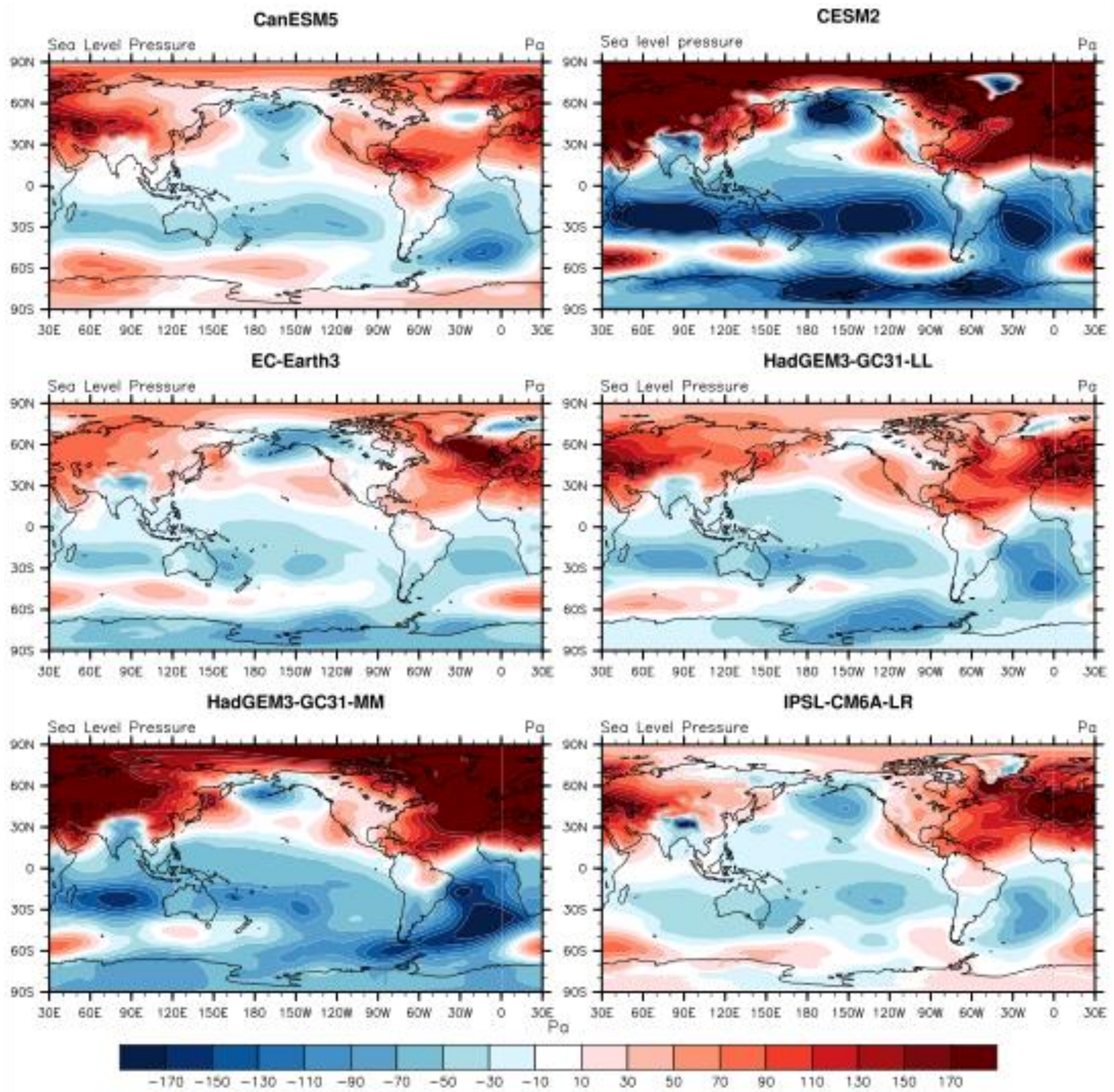

Fig. R2. AMOC impact of global sea level pressure (SLP). Annual mean SLP change (shading in hPa) during 51-100 year after hosing relative to preindustrial control for a CanESM5, b CESM2, c EC-Earth3, d HadGEM3-GC31-LL, e HadGEM3-GC31-MM and f IPSL-CM6A-LR from NAHosMIP.

Along with the distinct SST change between the two hemispheres, the SLP change (Fig. 4e in the original submission) also shows a general hemispheric increase and decrease in the Northern and Southern Hemispheres, respectively, as well as a sea level pressure decrease over the North Pacific around the location of the Aleutian Low.

To examine the robustness of our result, we look into the freshwater hosing experiments with 6 climate models from the North Atlantic Hosing Model Intercomparison Project (NAHosMIP). A uniform hosing is input into model ocean from 50°N to the Bering Strait with a hosing strength of 0.3 Sv to slow the AMOC (Jackson et al. 2003). In response to an AMOC slowdown, all models show a general sea level pressure increase and decrease in the Northern and Southern Hemispheres,

respectively. Most models also show a sea level pressure decrease over the North Pacific around the location of the Aleutian Low (Fig. R2). All these characteristics are consistent with our results for the difference between the free- and fixed-AMOC simulations.

Regarding the IVT, to our best knowledge, few studies explored its change in response to a weakened AMOC. This is perhaps the novelty of our study.

Jackson, L. C., Alastrué de Asenjo, E., Bellomo, K., Danabasoglu, G., Haak, H., Hu, A., Jungclaus, J., Lee, W., Meccia, V. L., Saenko, O., Shao, A., and Swingedouw, D. 2023. Understanding AMOC stability: the North Atlantic Hosing Model Intercomparison Project. Geoscientific Model Development, 16, 1975-1995.

Stouffer, R.J., Yin, J.J., Gregory, J.M., Dixon, K.W., Spelman, M.J., Hurlin, W., Weaver, A.J., Eby, M., Flato, G.M., Hasumi, H. and Hu, A., 2006. Investigating the causes of the response of the thermohaline circulation to past and future climate changes. Journal of Climate, 19, 1365-1387.

(3) Statement about AMOC weakening and increased AR frequency
Line 121–127 and Fig. 2: I still have some concerns about how a weakened AMOC increases AR frequency. Please demonstrate more clearly the causal chain "AMOC slowdown → circulation change → increased AR activity."
For the North Atlantic and other mid-latitude regions, the underlying mechanisms may differ. In the North Atlantic, local air–sea interactions and SST gradients may dominate, whereas in other regions teleconnections or jet-dynamics changes in the westerlies could play a larger role.

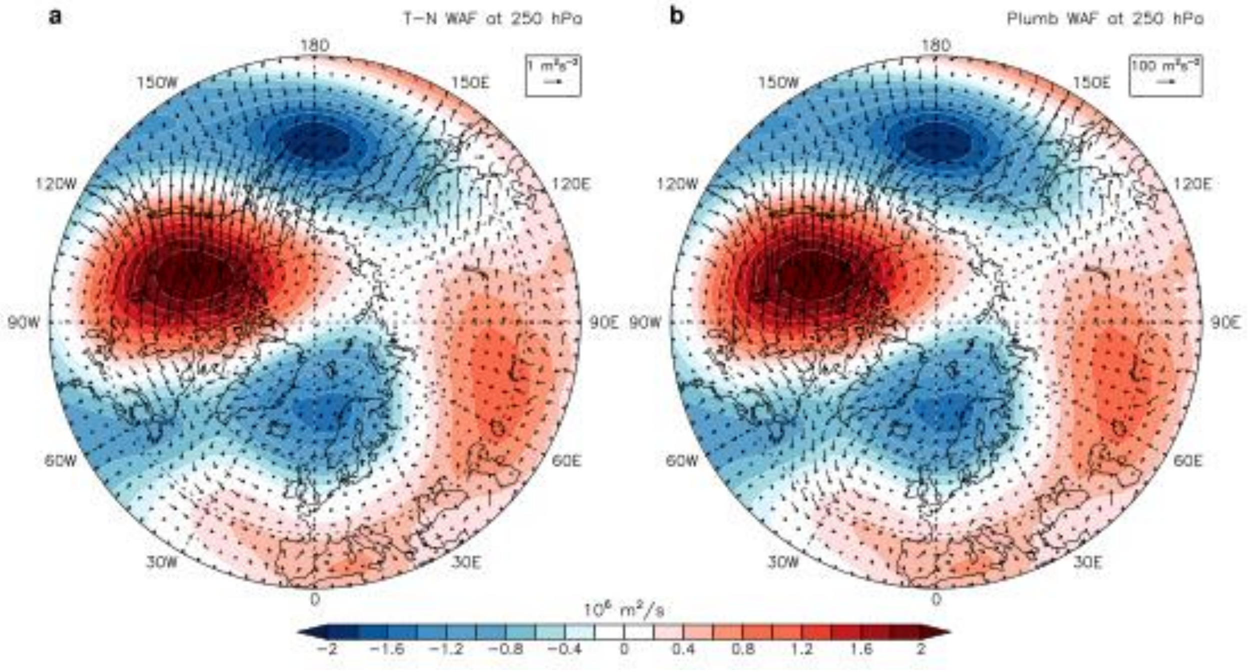

Fig. R3: AMOC impact on wave-activity flux. (a) Annual mean Takaya–Nakamura quasi-geostrophic streamfunction anomalies (shading in 10^6 m^2 s^{-1}), and horizontal wave-activity flux anomalies (vector in m^2 s^{-2}) associated with a stationary Rossby wave train at 250 hPa for the ensemble mean of CESM2 free- minus fixed-AMOC simulation during 2076–2100. (b) Same as (a)

but for quasi-geostrophic streamfunction anomalies and horizontal Plumb wave-activity flux anomalies.

We agree with the reviewer that local air-sea interactions and SST gradients play a significant role in atmospheric circulation change throughout the North Atlantic, and the AMOC impact can spread to other regions through atmospheric teleconnections. In the upper troposphere, the wave-activity flux is substantially divergent to the south of Greenland as a result of AMOC-induced surface cooling in the North Atlantic. It converges toward Euro-Asia before diverging again over the North Pacific, accompanied by anticyclonic and cyclonic anomalies in the "downstream development" (Fig. R3). The result is irrespective of whether Takaya–Nakamura or Plumb wave-activity flux is used.

We have included the above discussion and Fig. R3 in the revision.

Additionally, could the authors show global AR frequency changes based on observations or reanalysis data for validation?
Following the review, we show the linear trend in AR frequency over 1992–2014 from MERRA-2 reanalysis. We find a dipole pattern in the mid-latitudes with less and more frequent ARs in the equatorward and poleward sides, respectively, which is more prominent in the Southern Hemisphere. This pattern suggests a poleward shift of ARs as a response of external greenhouse gas forcing (Li and Ding 2024). Meanwhile, given the two-decade timescale (1992–2014), internal decadal variability may also play a role in such AR changes. Nonetheless, it is not straightforward to distinguish the AMOC impact on ARs from the aforementioned factors. That's why we conducted the fixed-AMOC experiment.

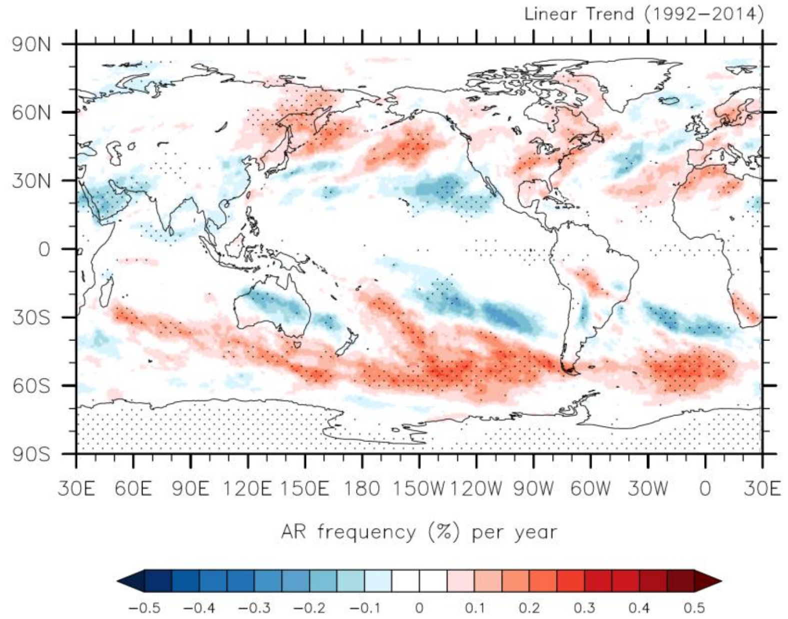

Fig. R4. MERRA-2 atmospheric river (AR) frequency. Linear trend in AR frequency (shading in % per year) over 1992–2014 from MERRA-2 reanalysis. Stippling marks areas where the trend is significant at the $p < 0.05$ level according to a two-tailed t-test.

Li, Z. and Ding, Q., 2024. A global poleward shift of atmospheric rivers. Science Advances, 10, eadq0604.

(4) Decomposition technique

The dynamic/thermodynamic decomposition is a useful first-order diagnostic, but it implicitly assumes linear separability and fixed circulation. Given that AMOC-induced temperature gradients can alter baroclinicity and atmospheric stability, this assumption may not hold. Please explain the potential limitations of this approach and how they affect your results.

We agree with the reviewer. The dynamic–thermodynamic decomposition provides a useful first-order diagnostic for interpreting AR responses, but with implicit assumptions of linear separability and fixed circulation. Given the complexity of the interaction between the thermal and dynamic parts such as altered temperature gradients and atmospheric circulation, assumptions may not fully hold for higher-order accuracy.

Nonetheless, even as a first-order diagnostic, the dynamic/thermodynamic decomposition captures the key physical drivers. For instance, Ma et al. (2021) showed that thermodynamic moisture increases often dominate AR changes, supporting the importance of separating moisture and circulation effects. By estimating the components separately using a scaling method, Ma et al. (2021) similarly found that the sum of the dynamic and thermodynamic contributions explains most of the AR response to Arctic sea-ice loss (see their Fig. 1a versus Fig. 1b), which suggests that the dynamic and thermodynamic components are largely linearly additive, i.e., the linear separability assumption is valid for decomposing AR changes into dynamic and thermodynamic contributions. Zhang et al. (2021) further demonstrated that AR response to global warming arises from both moisture increase and circulation adjustment. Our results are in line with these previous findings, showing that the decomposition captures the key mechanisms by which the slower AMOC modulates ARs, despite simplifications of the decomposition.

Following the review, we have added discussions on the potential limitations of this approach and its effect on our result to the revision.

Ma, W., Chen, G., Peings, Y., and Alviz, N., 2021. Atmospheric river response to Arctic sea ice loss in the Polar Amplification Model Intercomparison Project. Geophysical Research Letters, 48, e2021GL094883.

Zhang, P., Chen, G., Ma, W., Ming, Y. and Wu, Z., 2021. Robust atmospheric river response to global warming in idealized and comprehensive climate models. Journal of Climate, 34, 7717-7734.

(5) Physical mechanisms

Line 189: The manuscript attributes westerly-jet intensification to transient-eddy forcing and global teleconnections without providing diagnostics to support this claim. Including eddy momentum flux, E–P flux divergence, or storm-track activity maps would substantially strengthen the physical interpretation and demonstrate the mechanism driving AR changes.

Following the review, we have plotted the wave activity flux (see our reply to the previous comment), storm track (Fig. R5) and Eliassen–Palm (EP) flux (Fig. R6) for the ensemble mean of CESM2 free- minus fixed-AMOC simulation during 2076-2100. We infer storm tracks from the standard deviation of 2–6-day bandpass-filtered sea level pressure and find positive and negative anomalies to the north of 45°N and to the south of 45°S, respectively (Fig. R5). This result suggests that a weakened AMOC intensifies and reduces transient eddy activities in the mid-to high latitudes in the Northern and Southern Hemispheres, respectively.

We also look into eddy-mean flow interactions using the Eliassen–Palm (EP) flux vector containing contributions from both stationary and transient eddies, with its vertical component depicting the meridional heat flux and its meridional component depicting the equatorward meridional momentum flux, which is calculated using 25-year (2076-2100) daily data from free- minus fixed-AMOC simulation. In response to an AMOC slowdown, EP flux diverges between 45°N and 65°N, from surface to about 850 hPa, indicating that changes in eddy flux, primarily those from meridional eddy momentum flux, act to accelerate the low-level westerly winds over the region (Fig. R6). While EP flux converges above and to the south, acting to slow the mean flow there. It is worth noting that the EP flux anomalies should be understood as the positive feedback to, rather than the cause of, the change in the eddy-driven jet.

We have included the above discussions and Figs. R5 and R6 in the revision.

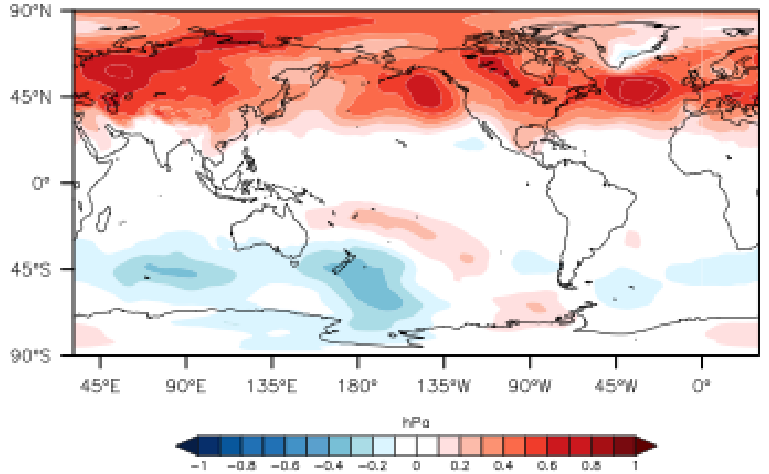

Fig. R5. AMOC impact on storm-track activity. Changes in storm track (shading in hPa) inferred from the standard deviation of 2–6-day bandpass-filtered sea level pressure for the ensemble mean of free- minus fixed-AMOC simulation during 2076–2100.

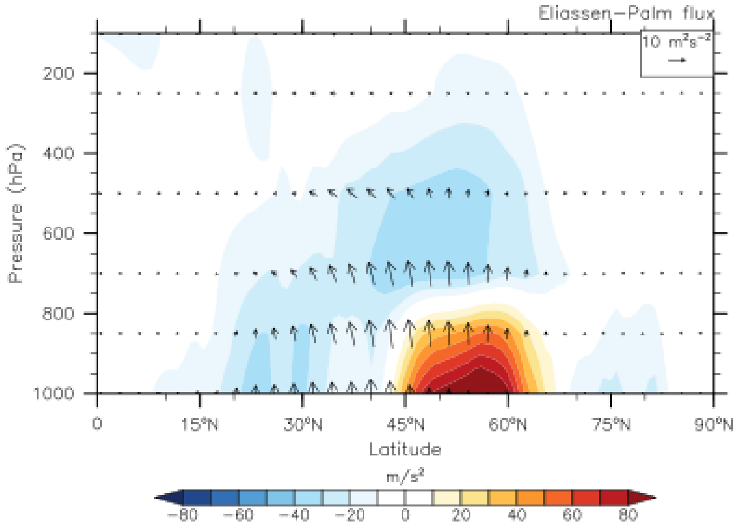

Fig. R6. AMOC impact on Eliassen–Palm (EP) flux. Annual mean EP flux (vectors in $m^2\ s^{-2}$) and its divergence (shading in $m\ s^{-2}$) calculated from daily data for the ensemble mean of CESM2 free- minus fixed-AMOC simulation during 2076-2100.

In addition, a schematic or conceptual cartoon summarizing the proposed mechanisms would be a valuable addition to the paper.
Thanks for the suggestion! We really wish we could, however, we lack professional art design for cartoon so have to skip the cartoon this time.

Minor Comments
Line 94: Please include the citation for CESM2. Is there any specific reason for choosing this model?
Sure. We have included the citation for CESM2 in this line in the revision. We choose CESM2 since it is the latest release in the CESM family of coupled Earth system models, with many science and infrastructure improvements. It is also a broadly used CMIP6-era model.

Figure 1: The figure appears to be of low quality; details are difficult to discern in the PDF version.
We have revised the figure and used the "eps" format in the revision.

Line 114: "less more frequent" should be corrected to "less frequent."
Revised as suggested.

Overall Recommendation
The manuscript presents a potentially valuable contribution to understanding AMOC–AR interactions under global warming. However, the physical reasoning and methodological transparency require further strengthening before publication. Addressing the above points—especially the experiment design justification and the diagnostic evidence for dynamic mechanisms—would significantly enhance the paper's scientific rigor and clarity.

Reviewer #3 (Remarks to the Author):

The manuscript needs substantial revision. Below are my comments

Thanks for the insightful advice and comments!

Following reviewer's comment, we use a common historical (1990-2014) climatology from CESM2 free simulation to analyze the 85th percentile of IVT and ARs in all the simulations for all the time periods. Thus, we amend Figs. 2, 3 and 4, as well as corresponding supplementary figures, but our main finding remains unchanged, indicating that the choice of baseline climatology to compute IVT and AR has little effect on our conclusions of AMOC impacts on ARs and AR-induced precipitation.

Major comments:

1. The manuscript relies heavily on the contrast between free-AMOC and fixed-AMOC simulations. However, the methodological description of the freshwater extraction strategy (page 10–11) lacks quantitative details:

How much freshwater is removed annually? How sensitive is the AMOC stabilization to spatial pattern and magnitude of removal?

Following the review, the methodological description of the freshwater extraction strategy has been added to the "Method" section.

2. Could unintended circulation/stratification artifacts occur in the subpolar gyre?

The AMOC weakening will bring about oceanic adjustments, for example in CCSM4, to decelerate the subpolar gyre circulation in the North Atlantic, which, involves atmospheric response and ocean-atmosphere interaction in that region (Mimi and Liu 2024). This is also true for the current CESM2 experiments (Fig. R7).

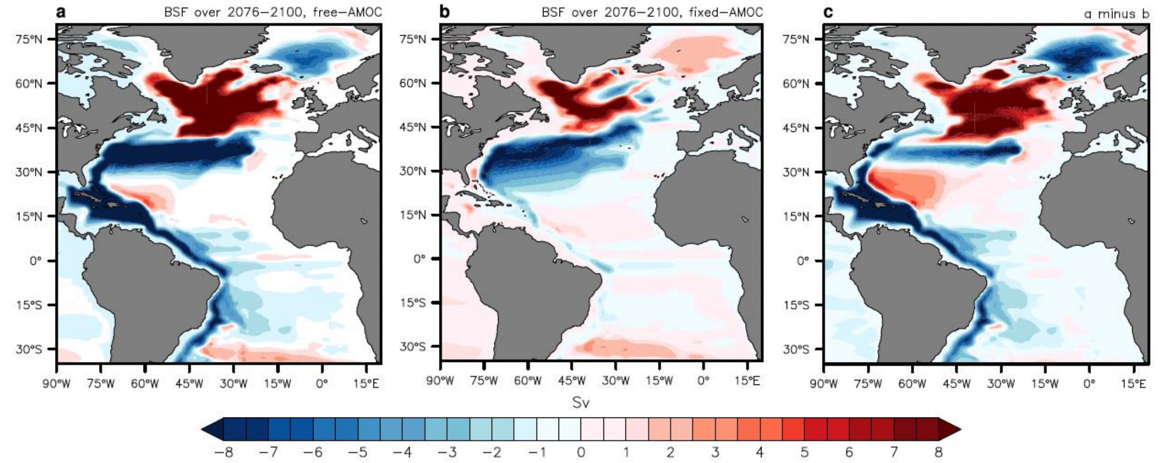

Fig. R7. AMOC impact on Atlantic gyre circulation change. Annual mean changes in barotropic stream function (color in Sv) for the ensemble means of CESM2 (a) free- and (b) fixed-AMOC simulations during 2076–2100 relative to 1990–2014, and (c) the difference between the two (a minus b).

3. A clearer justification and a sensitivity demonstration are essential, since the entire attribution of AMOC impacts depends on this experimental setup.

Yes, we clarify the scheme of freshwater removal for CESM2 in the revision, which is consistent with our previous scheme for CCSM4. The magnitude and timings differ between CESM2 and CCSM4, which is due to model difference.

We don’t have sufficient computation resources to reconduct our fixed-AMOC experiments for a sensitivity test, for example, changing the area of freshwater removal. Nevertheless, we have looked at CESM2 experiment from the NAHosMIP (Jackson et al. 2003), in which a uniform hosing of 0.3 Sv was input from 50°N to the Bering Strait (including the Arctic). The result of the NAHosMIP, for example, SLP change (Fig R2), is consistent with our CESM2 experiment result.

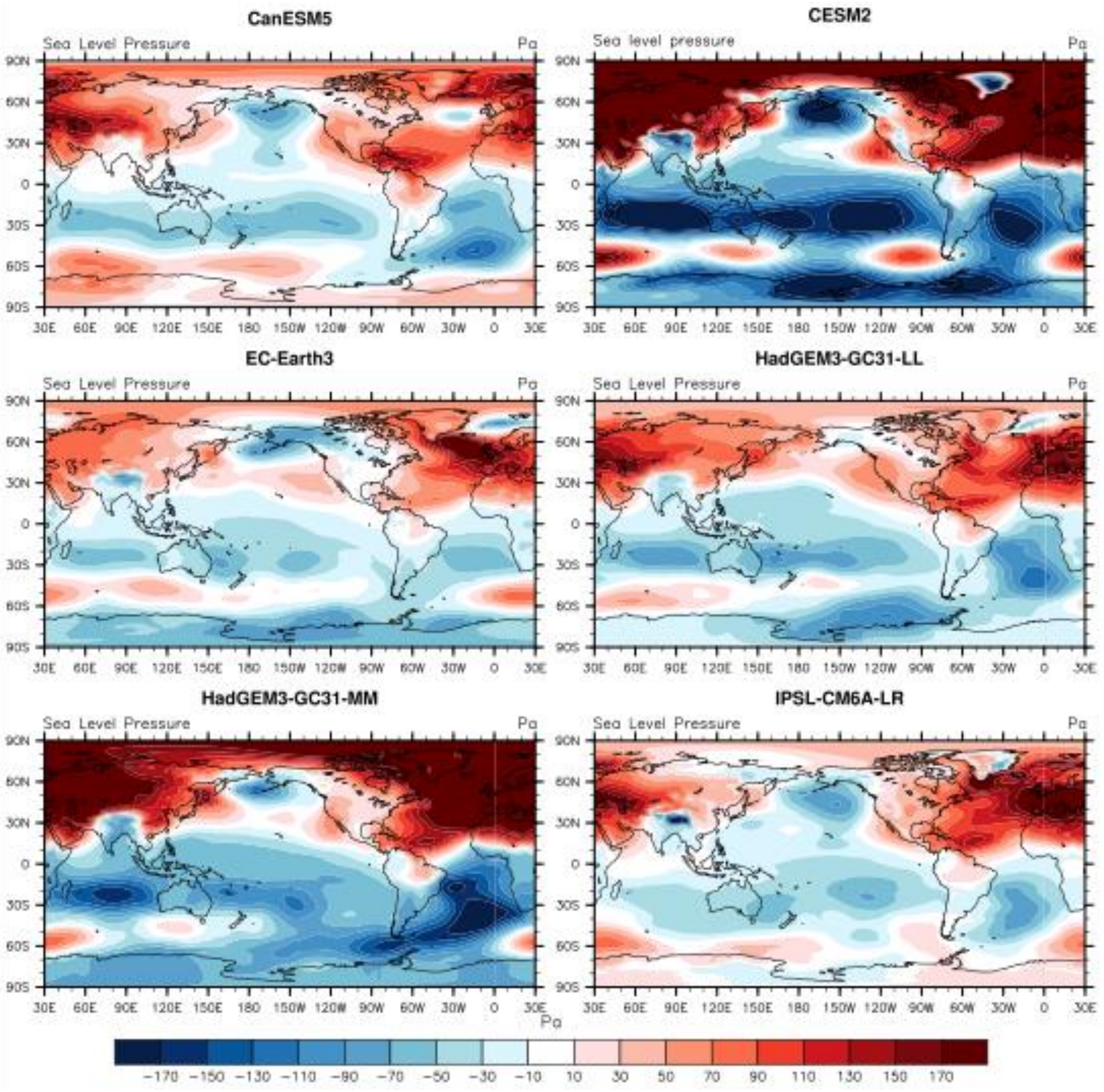

Fig. R2. AMOC impact of global sea level pressure (SLP). Annual mean SLP change (shading in hPa) during 51-100 year after hosing relative to preindustrial control for a CanESM5, b CESM2, c EC-Earth3, d HadGEM3-GC31-LL, e HadGEM3-GC31-MM and f IPSL-CM6A-LR from the NAHosMIP.

4. Although MERRA-2 ARs are used as a benchmark, the manuscript does not present a systematic evaluation of CESM2's fidelity in AR characteristics (frequency, IVT distribution, seasonal cycle, landfall latitudes). Given known issues in coarse-resolution AR representation (especially jet latitude biases, storm-track spread), readers need at least one figure/table comparing CESM2 vs. MERRA-2 or other ARTMIP datasets. This is crucial before inferring robust future changes.
Following the review, we have compared AR characteristics between MERRA-2 and CESM2 free-AMOC simulation from 1990 to 2014. We find that CESM2 can generally well simulate the AR characteristics, including IVT distribution, AR frequency (Fig. R8), seasonal cycle (Fig. R9) and landfall latitudes (Fig. R10). This result demonstrates CESM2's fidelity in AR simulation, despite the model's relatively coarse resolution compared to high-resolution models.

We have added Figs. R8-R10 and the above discussion to the revision.

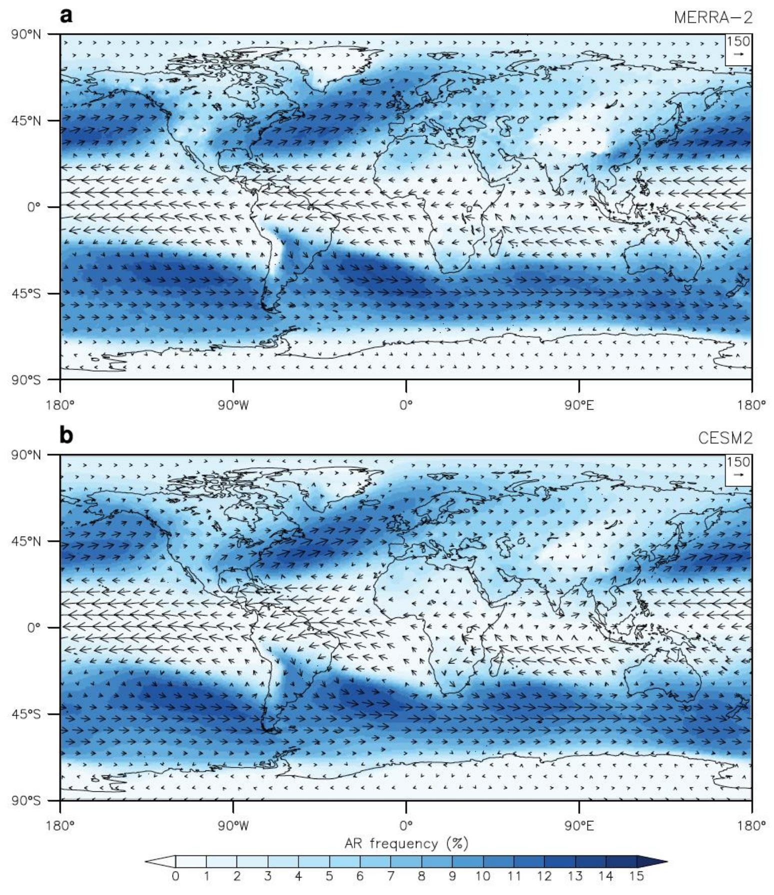

Fig. R8. AR frequency and IVT in reanalysis and CESM2. (a) Annual mean climatological AR frequency (shading in %) with IVT (vectors in kg/m/s) derived from NASA MERRA-2 reanalysis data over 1990-2014 period. (b) Same as a but for the ensemble mean of CESM2 free-AMOC simulation over the same period.

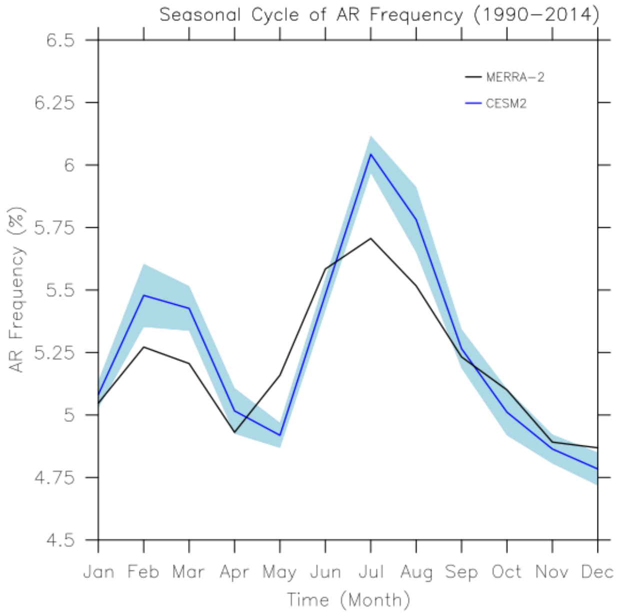

Fig.R9. Seasonal cycle of AR frequency in reanalysis and CESM2. Monthly and Global mean AR frequency averaged over 1990–2014 from NASA MERRA-2 reanalysis (black) and CESM2 free-AMOC simulation (ensemble mean, blue; ensemble spread, light blue). CESM2 ensemble spread is calculated as one standard deviation among ensembles.

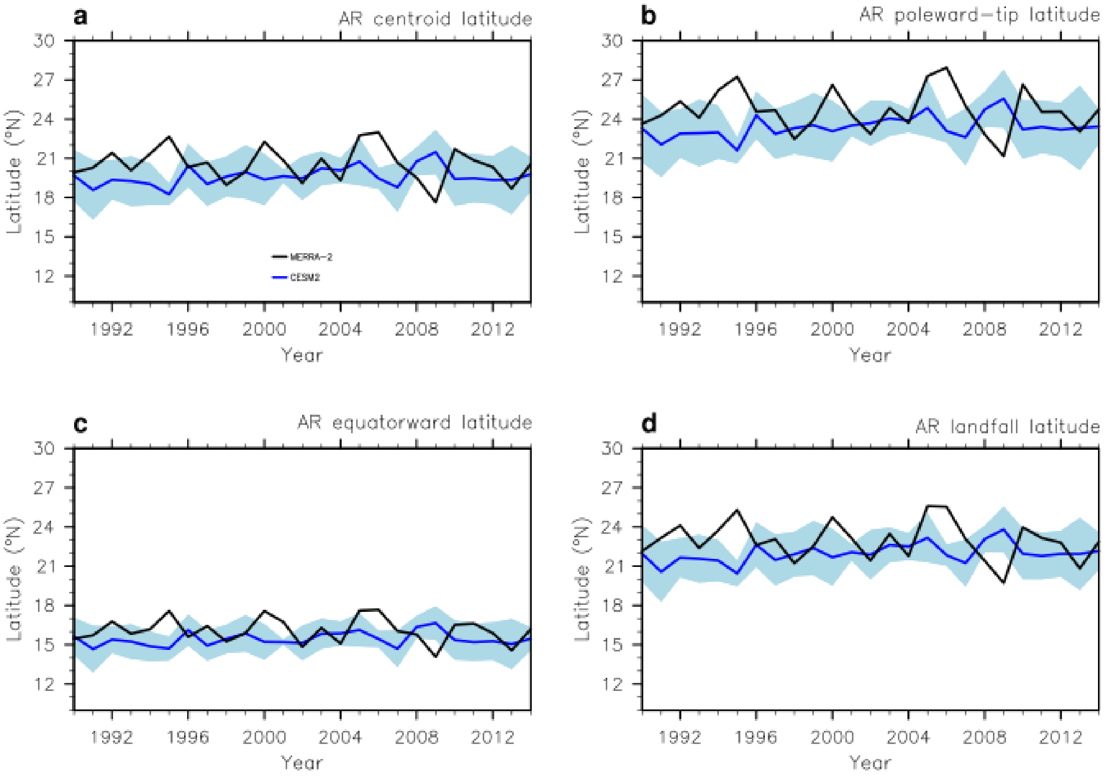

Fig. R10. AR latitudes in reanalysis and CESM2. (a) Annual mean AR centroid latitude, (b) annual mean AR poleward-tip latitude, (c) annual mean AR equatorward-tip latitude, and (d) annual mean AR landfall latitude over 1990–2014 from NASA MERRA-2 reanalysis (black) and

CESM2 free-AMOC simulation (ensemble mean, blue; ensemble spread, light blue). Latitudes are averaged across all detected AR events in each year. CESM2 ensemble spread is calculated as one standard deviation among ensembles.

5. The dynamic response figure (page 22) shows a strong intensification of Pacific westerlies under AMOC slowdown. However, the mechanisms remain vaguely described. Is this a teleconnection via stationary waves (as suggested in Liu et al. 2020)?
How do SST anomalies propagate from the North Atlantic into the Pacific jet region? What is the relative contribution of transient eddies vs. mean-flow adjustment?
A more mechanistic, process-level explanation—possibly including zonal-mean wind anomalies or wave activity flux—would significantly strengthen the paper.

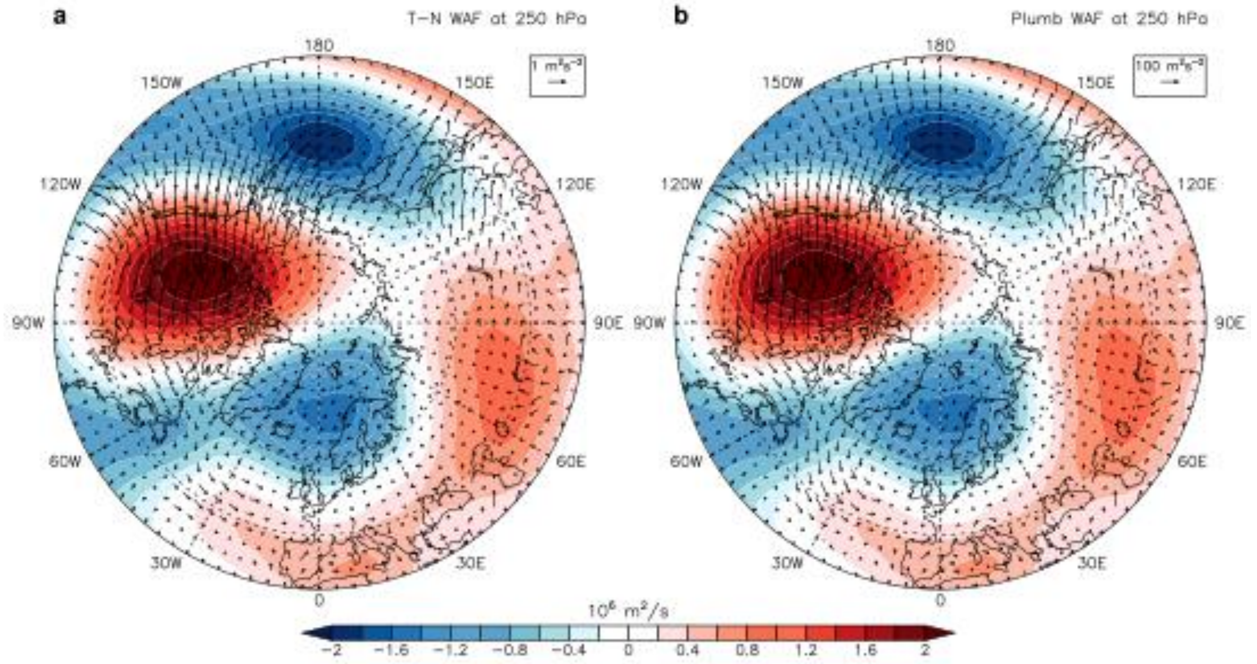

Fig. R3: AMOC impact on wave-activity flux. (a) Annual mean Takaya–Nakamura quasi-geostrophic streamfunction anomalies (shading in 10^6 m^2 s^{-1}), and horizontal wave-activity flux anomalies (vector in m^2 s^{-2}) associated with a stationary Rossby wave train at 250 hPa for the ensemble mean of CESM2 free- minus fixed-AMOC simulation during 2076–2100. (b) Same as (a) but for quasi-geostrophic streamfunction anomalies and horizontal Plumb wave-activity flux anomalies.

We agree with the reviewer that local air-sea interactions and SST gradients play a significant role in atmospheric circulation change throughout the North Atlantic, and the AMOC impact can spread to other regions through atmospheric teleconnections. In the upper troposphere, the wave-activity flux is substantially divergent to the south of Greenland as a result of AMOC-induced surface cooling in the North Atlantic. It converges toward Euro-Asia before diverging again over the North Pacific, accompanied by anticyclonic and cyclonic anomalies in the "downstream development" (Fig. R3). This pattern is similar to that shown by Liu et al. (2020). Note that our result is irrespective of whether Takaya–Nakamura or Plumb wave-activity flux is used.

We have added Fig. R3 and the above discussion to the revision.

Minor comments:
1. The 85th percentile IVT threshold is recomputed separately for 1990–2014 and 2076–2100 (page 10). This may artificially shift AR frequency due to changing climatological moisture. Authors should comment on how this choice affects comparability.
Following the review, we adopt the same historical climatology of IVT threshold during 1990-2014 from the free-AMOC simulation for all the analysis to avoid the issue as the reviewer mentioned. Applying a consistent historical threshold facilitates fair comparisons across periods and experiments and captures the combined effects of moisture and circulation changes on the total AR response. As shown in the revised manuscript, our main conclusion still holds after this change.

2. Some figures (e.g., dynamic/thermodynamic responses on page 22) lack clear unit notation in captions. Units for frequency change (%) vs. absolute frequency need explicit distinction.
Revised as suggested.

3. The zonal means on the right panels (pages 20–21) are difficult to interpret due to compressed axis scaling. Consider rescaling axes or enlarging the side panels for clarity.
Revised as suggested. We have also changed the figure format to "eps" for better visibility.

Reviewer #1 (Remarks to the Author):

The current version of the manuscript provides significant analyses on the impact of the Atlantic meridional overturning circulation (AMOC) on atmospheric rivers (ARs) throughout the twenty-first century. The authors have answered most of my concerns.

1.1 Major Comments
With minor revisions, the manuscript could improve our understanding of the interactions between the AMOC and the AR in a warming climate. The authors found that weakened AMOC increases AR frequency in mid-latitudes by intensifying the prevailing westerly winds, particularly along the West Coast of North America.

• What is still missing is how an increase in AR frequency impacts the West Coast of North America, for example. Corringham et al. suggested that ARs are responsible for the majority of economic losses from flooding in the Western United States and are expected to intensify in a warming climate. They argued that flood damage from extreme AR events may triple, from 1 billion to over 3 billion a year, toward the end of the century if mitigation actions are not implemented to reduce anthropogenic greenhouse gas emissions.

Can the author examine or discuss how he by 0.18 m/year (up to 0.44 m/year) enhance AR-induced precipitation of 0.18 m/year (up to 0.44 m/year) caused by the weakened AMOC will affect the US West Coast?

Corringham TW, McCarthy J, Shulgina T, Gershunov A, Cayan DR, Ralph FM. Climate change contributions to future atmospheric river flood damages in the western United States. Nature Scientific Reports. 2022;12:13747. DOI: 10.1038/s41598-022- 15474-2

Yes, as shown in our Figs. 2 and 3, the AMOC slowdown contributes to changes in AR frequency and AR-induced precipitation at the US West Coast, while it still need bear in mind that the AR frequency and AR-induced precipitation are also strongly modulated by background greenhouse gas emissions. Therefore, we have cited the study by Corringham et al. in the revision to clarify that the AMOC-influenced AR-induced precipitation will also contribute to flood damage at the US West Coast.

• The section about the Eliassen-Palm flux and the Takaya-Nakamura and Plumb wave- activity fluxes needs more details. The section about the Eliassen-Palm flux and the Takaya-Nakamura and Plumb wave-activity fluxes needs more details. In equations 6 (line 413), 7 (line 426), and 8 (line 431), how the perturbation stream-function or the departures from the zonal means were computed. Did the authors use a turbulence closure scheme?
I recommend minor revision before further publication.

For one variable (such as u, v, etc.), we first calculate its zonal mean and then subtract the zonal mean from this variable to get the departure from the zonal mean.

Comments
1-Line 86-88. Change all occurrences of "observed" to reanalysis. The text in line 87 is confusing. Do the authors mean " We show the evolution of AR...."
Revised as suggested.

2-Line 184. "diminished and enhances" they represent opposing action, please clarify.
The slower AMOC diminishes the likelihood of the mean IVT in azimuths of smaller than 90° and enhances the likelihood of the mean IVT in azimuths of larger than 90°. We used "respectively" in the end to clarify this.

3-Line 298. "... the decelerated Atlantic overturning" circulation is missing.
Atlantic overturning is a brief expression for Atlantic meridional overturning circulation. In response to the review, we have revised it to "the decelerated AMOC".

4- Line 473-475. Please rephrase. I suggest : internal; climate variability can influence AR's ....
Revised as suggested.

2.1 Figures.
The figures need improvement before publication (Fig 2, Fig 3, Fig 4, Fig 6). The figures at the end of the manuscript are better compared to those in within the manuscript. I will suggest to increase the font size on the axis.
Caption of multipanel figure. For all the figure in the supplementary, please add a comma, a bracket or a colon between the letter identifying the panel and the text. For example, in Figures 1,2, replace "a Annual" by "a, Annual"
Thanks for the suggestion. Meanwhile, the figures and figure captions need to follow the format of the journal.

Atlantic meridional overturning circulation slowdown modulates atmospheric rivers in a warmer climate

1 Overview

The current version of the manuscript provides significant analyses on the impact of the Atlantic meridional overturning circulation (AMOC) on atmospheric rivers (ARs) throughout the twenty-first century. The authors have answered most of my concerns.

1.1 Major Comments

With minor revisions, the manuscript could improve our understanding of the interactions between the AMOC and the AR in a warming climate. The authors found that weakened AMOC increases AR frequency in mid-latitudes by intensifying the prevailing westerly winds, particularly along the West Coast of North America.

- What is still missing is how an increase in AR frequency impacts the West Coast of North America, for example. Corringham et al. suggested that ARs are responsible for the majority of economic losses from flooding in the Western United States and are expected to intensify in a warming climate. They argued that flood damage from extreme AR events may triple, from 1 billion to over 3 billion a year, toward the end of the century if mitigation actions are not implemented to reduce anthropogenic greenhouse gas emissions.

  Can the author examine or discuss how he by 0.18 m/year (up to 0.44 m/year) enhance AR-induced precipitation of 0.18 m/year (up to 0.44 m/year) caused by the weakened AMOC will affect the US West Coast?

  Corringham TW, McCarthy J, Shulgina T, Gershunov A, Cayan DR, Ralph FM. Climate change contributions to future atmospheric river flood damages in the western United States. Nature Scientific Reports. 2022;12:13747. DOI: 10.1038/s41598-022-15474-2

- The section about the Eliassen-Palm flux and the Takaya-Nakamura and Plumb wave-activity fluxes needs more details. The section about the Eliassen-Palm flux and the Takaya-Nakamura and Plumb wave-activity fluxes needs more details. In equations 6

(line 413), 7 (line 426), and 8 (line 431), how the perturbation stream-function or the departures from the zonal means were computed. Did the authors use a turbulence closure scheme?

I recommend minor revision before further publication.

2 Comments

1-Line 86-88. Change all occurrences of "observed" to reanalysis. The text in line 87 is confusing. Do the authors mean " We show the evolution of AR...."
2-Line 184. "diminished and enhances" they represent opposing action, please clarify.
3-Line 298. "... the decelerated Atlantic overturning" circulation is missing.
4- Line 473-475. Please rephrase. I suggest : internal; climate variability can influence AR's ....

2.1 Figures.

The figures need improvement before publication (Fig 2, Fig 3, Fig 4, Fig 6). The figures at the end of the manuscript are better compared to those in within the manuscript. I will suggest to increase the font size on the axis.

Caption of multipanel figure. For all the figure in the supplementary, please add a comma, a bracket or a colon between the letter identifying the panel and the text. For example, in Figures 1,2, replace "a Annual" by "a, Annual"
